# Gene expression and activity of digestive proteases in *Daphnia*: effects of cyanobacterial protease inhibitors

**DOI:** 10.1186/1472-6793-10-6

**Published:** 2010-05-04

**Authors:** Anke Schwarzenberger, Anja Zitt, Peter Kroth, Stefan Mueller, Eric Von Elert

**Affiliations:** 1 University of Cologne, Cologne Centre for Biosciences, Zülpicher Straße 47 b, 50674 Cologne, Germany; 2 University of Konstanz, 78457 Konstanz, Germany; 3 University of Cologne, Centre for Molecular Medicine Cologne, 50931 Cologne, Germany

## Abstract

**Background:**

The frequency of cyanobacterial blooms has increased worldwide, and these blooms have been claimed to be a major factor leading to the decline of the most important freshwater herbivores, i.e. representatives of the genus *Daphnia*. This suppression of *Daphnia *is partly attributed to the presence of biologically active secondary metabolites in cyanobacteria. Among these metabolites, protease inhibitors are found in almost every natural cyanobacterial bloom and have been shown to specifically inhibit *Daphnia*'s digestive proteases *in vitro*, but to date no physiological responses of these serine proteases to cyanobacterial protease inhibitors in *Daphnia *have been reported *in situ *at the protein and genetic levels.

**Results:**

Nine digestive proteases were detected in *D. magna *using activity-stained SDS-PAGE. Subsequent analyses by LC-MS/MS and database search led to the identification of respective protease genes. *D. magna *responded to dietary protease inhibitors by up-regulation of the expression of these respective proteases at the RNA-level and by the induction of new and less sensitive protease isoforms at the protein level. The up-regulation in response to dietary trypsin- and chymotrypsin-inhibitors ranged from 1.4-fold to 25.6-fold. These physiological responses of *Daphnia*, i.e. up-regulation of protease expression and the induction of isoforms, took place even after feeding on 20% cyanobacterial food for only 24 h. These physiological responses proved to be independent from microcystin effects.

**Conclusion:**

Here for the first time it was shown *in situ *that a *D. magna *clone responds physiologically to dietary cyanobacterial protease inhibitors by phenotypic plasticity of the targets of these specific inhibitors, i.e. *Daphnia *gut proteases. These regulatory responses are adaptive for *D. magna*, as they increase the capacity for protein digestion in the presence of dietary protease inhibitors. The type and extent of these responses in protease expression might determine the degree of growth reduction in *D. magna *in the presence of cyanobacterial protease inhibitors. The rapid response of *Daphnia *to cyanobacterial protease inhibitors supports the assumption that dietary cyanobacterial protease inhibitors exert a strong selection pressure on *Daphnia *proteases themselves.

## Background

Increasing nutrient input has led to eutrophication in many lakes, which coincides with the increasing dominance of bloom-forming cyanobacteria in the phytoplankton assemblages [1,2]. This increasing dominance of cyanobacteria has been claimed to be a major factor leading to the decline in *Daphnia *abundance across and within lakes [3-5]. These observations from the field are corroborated by laboratory studies which have demonstrated negative effects of cyanobacteria on *Daphnia *[6,7]. However, the generality of these observations has been questioned by a manipulative field study [8], and other recent studies have indicated that *Daphnia *may adapt to increasingly tolerate dietary cyanobacteria [9-11] and that increased tolerance to cyanobacterial toxins may be transferred to the offspring generations [12]. However, neither on protein nor on genetic level have the underlying mechanisms for increased tolerance been addressed, yet. The genome of *Daphnia *has recently become available, creating the opportunity to address the interaction of cyanobacteria and *Daphnia *on the levels of gene expression and proteins more specifically.

Cyanobacteria are known to contain toxins and an array of other biologically active secondary metabolites [13,14]. Cyanobacterial protease inhibitors are among the most widely spread secondary metabolites, as they have been found in nearly every cyanobacterial bloom [14,15]. Different protease inhibitors have been isolated from different cyanobacteria genera [16] as well as from different cyanobacterial strains of the same species [17,18]. Many cyanobacterial protease inhibitors act against serine proteases (i.e. trypsins and chymotrypsins), which represent the most important digestive proteases in the gut of *Daphnia magna *[19], and of which a surprisingly high number was found in the genome of *D. pulex *[20], a closely related species of *D. magna*.

Here we tested whether *Daphnia *displays physiological plasticity in response to dietary cyanobacterial protease inhibitors, assuming that positive selection for increased plasticity might be one mechanism for the recently reported adaptation of *Daphnia *to co-occuring cyanobacteria [21]. More specifically, we investigated the physiological response of a given genotype of *D. magna *to dietary cyanobacterial protease inhibitors, making use of the previously reported specific interaction of these inhibitors with digestive trypsins and chymotrypsins of *D. magna *[19]. By feeding them mixtures of a green alga with two different strains of the cyanobacterium *Microcystis aeruginosa*, which either contained trypsin- or chymotrypsin inhibitors, we were able to independently determine the effects of the two families of inhibitors on the expression and the activity of *D. magna*'s digestive proteases.

## Results

### Somatic growth rates on different food treatments

**Figure 1:**
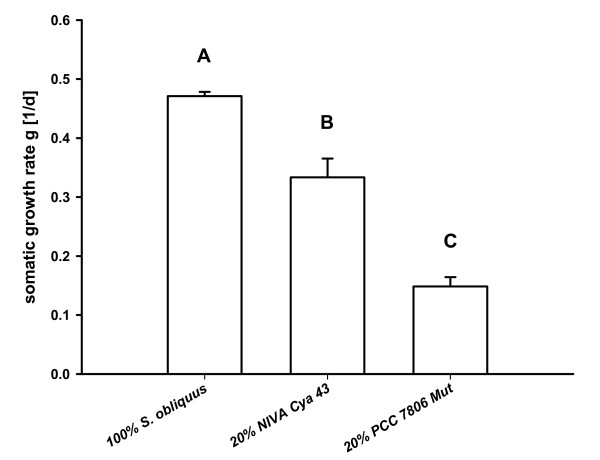
**somatic growth rates**. Mean somatic growth rates of *Daphnia magna *grown on either 100% *S. obliquus*, 20% *M. aeruginosa *NIVA Cya 43 (with chymotrypsin inhibitors) and 80% *S. obliquus *or on 20% *M. aeruginosa *PCC 7806 Mut (with trypsin inhibitors) and 80% *S. obliquus *(n = 3, ± SD). Letters indicate a significant (p < 0.05) difference between the treatments.

When feeding on the green alga *S. obliquus*, *D. magna *grew at 0.47 d^-1 ^(Fig. 1), whereas the growth rate on 20% of the cyanobacterium *M. aeruginosa *was significantly reduced (one way ANOVA: p < 0.05; F_2,6 _= 180.8) in both cyanobacterial treatments. Growth on the mutant of PCC 7806 was significantly lower (0.15 d^-1^) than on NIVA Cya 43 (0.33 d^-1^).

### Activity and stability of *Daphnia *serine proteases

The chymotrypsin activity of the *Daphnia *homogenate was 0.23 μmol pNA/min/μg protein. There was a small, albeit significant (one way ANOVA: p < 0.05; F_1,4 _= 0.0002) decrease between the specific activity of chymotrypsin of the *Daphnia *homogenate and the same homogenate treated with 2 M urea (0.22 μmol pNA/min/μg protein).

The specific trypsin activity of the *Daphnia *homogenate was 0.02 μmol pNA/min/μg protein. There was no significant (one way ANOVA: p = 0.06; F_1,4 _= 0.06) effect of 2 M urea on trypsin activity.

### SDS-PAGE and native PAGE of *Daphnia *homogenate

**Figure 2:**
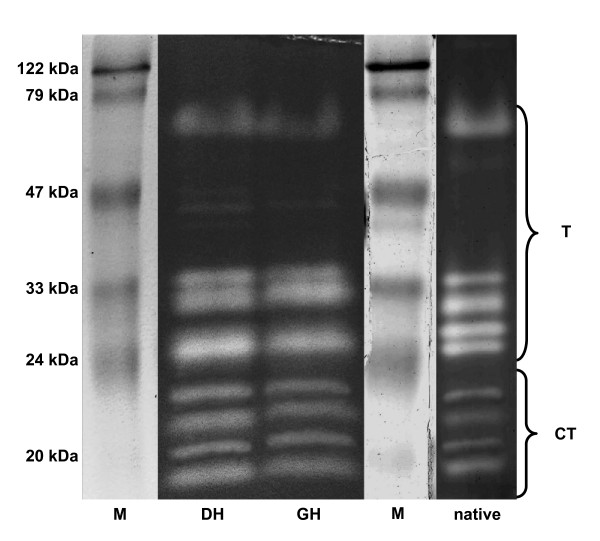
**SDS-PAGE and native gel of *Daphnia *homogenate**. Activity stained SDS-PAGE of *Daphnia *homogenate (DH) or gut homogenate (GH) and a native PAGE of *Daphnia *homogenate. White bands indicate active proteases. Protein marker (M). Trypsins (T) and chymotrypsins (CT) are assigned according to Agrawal et al., 2005 [19].

The protease pattern (Fig. 2) of the homogenate of *D. magna *grown on 100% *S. obliquus *showed five bands for trypsins (between 24 - 70 kDa; [19]) and four bands (between 18 - 23 kDa) which, based on indirect evidence, had been previously suggested to be chymotrypsins [19]). There was no difference between the protease pattern of the *Daphnia *homogenate (whole *Daphnia*) and the gut homogenate (gut + hepatopancreas). The pattern was the same on a native PAGE (Fig. 2).

### Food treatments: SDS-PAGE

**Figure 3:**
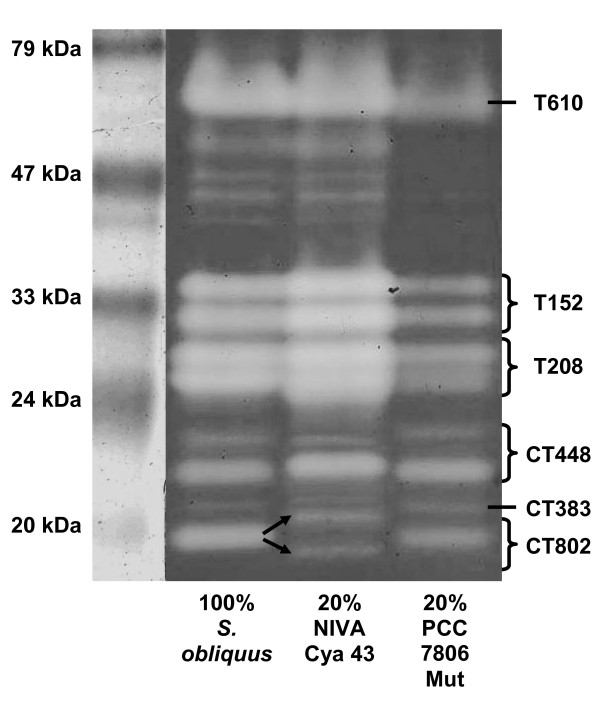
**SDS-PAGE of homogenate of *D. magna *grown on different food treatments**. Activity stained SDS-PAGE of homogenate of *D. magna *grown on either 100% *S. obliquus*, 20% *M. aeruginosa *NIVA Cya 43 (with chymotrypsin inhibitors) and 80% *S. obliquus*, or on 20% *M. aeruginosa *PCC 7806 Mut (with trypsin inhibitors) and 80% *S. obliquus*. White bands indicate active proteases. Arrows point at shifts in the protease pattern. The numbers to the right depict the results of LC-MS/MS analysis and database search of the proteases (CT = chymotrypsin, T = trypsin).

The protease pattern (Fig. 3) of the homogenate of *D. magna *grown on 100% *S. obliquus *was the same as that of the homogenate of *D. magna *grown on 20% of the microcystin-free mutant of the cyanobacterium PCC 7806 (containing mostly trypsin inhibitors) [19] with regard to the number and the apparent molecular weight of the bands. However, weaker trypsin bands in the treatment with the cyanobacterium indicated reduced trypsin activity, while the intensity of the chymotrypsin bands was not affected.

In the homogenate of *D. magna *grown on 20% NIVA Cya 43 (which contains strong chymotrypsin inhibitors [17]), the band pattern of the trypsins with regard to the number and the apparent molecular weight of the bands did not change in comparison to animals grown on pure 100% *S. obliquus*. However, the intensity of the trypsin bands between 24 and 34 kDa increased; as did the chymotrypsin band at 21 kDa. A different band pattern in the chymotrypsin bands also became obvious (Fig. 3). The two visible bands between 17 and 19 kDa on the SDS-PAGE in the treatment with the cyanobacterium had a different apparent molecular weight than the 18 kDa chymotrypsin band in the treatment with only the green alga.

### Amplification efficiencies of the protease primers

The amplification efficiencies (AE) of the protease QPCR-primers all had a value around 1 (Table 1), which means a doubling of DNA in every cycle. The amplification efficiencies were considered in the analysis of the QPCR results.

**Table 1:** amplification efficiencies

Serine protease	AE
T152	1.0472
T208	1.0001
CT383	1.0452
CT448	1.0525
CT802	1.0554

The amplification efficiencies (AE) of the primers developed for QPCR of the five serine proteases.

### Food treatments: QPCR

**Figure 4:**
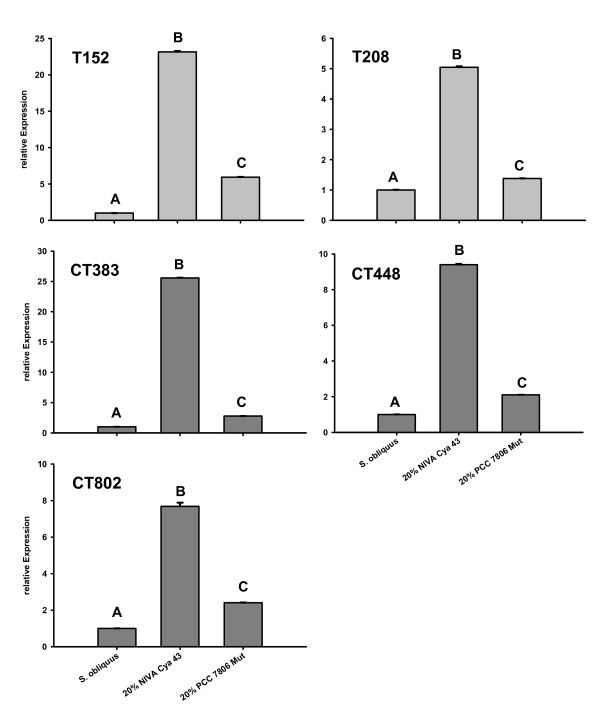
**relative gene expressions of proteases of *D. magna *grown on different food treatments**. *D. magna *were grown on three different food treatments (100% *S. obliquus*, 20% *M. aeruginosa *NIVA Cya 43 (with chymotrypsin inhibitors) and 80% *S. obliquus *or on 20% *M. aeruginosa *PCC 7806 Mut (with trypsin inhibitors) and 80% *S. obliquus*). Depicted is the mean relative expression (n = 3, ± SD) of trypsins (T152, T208) or chymotrypsins (CT448, CT383, CT802). Letters indicate a significant difference (p < 0.05) between calibrator (*S. obliquus*) and cyanobacterial food treatments.

The treatment with 100% *S. obliquus *served as the calibrator for the other treatments; its relative protease expression was therefore always set to 1. *Alpha-tubulin*, *SucDH *and *GapDH *served as endogenous controls. The expression of the proteases changed significantly in both cyanobacterial treatments (one way ANOVA: ***T152*: **F_2,8 _= 210813.1; p < 0.01; ***T208*: **F_2,6 _= 62182.1 p < 0.01; ***CT383*: **F_2,6 _= 1141713.5; p < 0.01; ***CT448*: **F_2,6 _= 229315.3; p < 0.01; ***CT802*: **F_2,8 _= 2455.6; p < 0.01) compared to the calibrator (Fig. 4).

In the treatment with 20% NIVA Cya 43, the proteases were up-regulated between 5.05-fold (*T208*) and 26.7-fold (*CT383*); in the treatment with 20% PCC 7806 Mut the relative expression was also significantly up-regulated in all proteases; however, the effects were weaker than in the treatment with NIVA Cya 43 [between 1.4-fold (*T208*) and 5.9-fold (*T152*)]; (Fig. 4).

### LC-MS/MS analysis of protease bands

The homogenate of *Daphnia *fed with 100% *S. obliquus *and the homogenate of *Daphnia *fed with NIVA Cya 43 (leading to a different protease band pattern) (Fig. 3) were subjected to LC-MS/MS analysis. Most bands of the SDS-PAGE with homogenate of *D. magna *fed with 100% *S. obliquus *could be identified via LC-MS/MS and database search using the MOWSE algorithm as implemented in the MS search engine Mascot (Matrix Science Ltd. London, UK) [22] (Additional file 1). The sequence match for the mass spectra of the bands from the 100% *S. obliquus *homogenate was low (≤ 10%). However, there were hardly any strikingly non-matching sequences. Since the proteases had very few cutting sites for trypsins, which is not surprising for they are all serine proteases, the sequence match with 7 to 10% was acceptable.

The bands of the 100% *S. obliquus *homogenate previously suggested to be chymotrypsins [19] could be assigned to the proteases 802 (18 kDa band) and 448 (22 kDa and 21 kDa bands), which had the best combination of the factors probability MOWSE score as well as the highest number of matched sequences and sequence coverage (Additional file 1). All three identified proteases were chymotrypsins. Only one (20 kDa) of the suggested chymotrypsin bands could not be identified. The bands between 24 and 34 kDa, formerly specified as trypsins [19], could be assigned to the proteases 152 and 208, which are both trypsins. They could not be differentiated because they matched to the same set of sequences.

However, the bands of the homogenate of *D. magna *fed with 20% NIVA Cya 43 and 80% *S. obliquus *(Fig. 3) could all be identified (Additional file 2). The LC-MS/MS results of the bands between 24 and 34 kDa were identical to those of the respective bands in the 100% *S. obliquus *lane and were assigned to the trypsins 152 and 208. However, here the identification was clearer: the two bands at 24 and 25 kDa were trypsin 208; the two others were trypsin 152. The band at 75 kDa that was visible in both gels could be identified as protease 610 in the 20% NIVA Cya 43 homogenate, which is also a trypsin, matching the results of Agrawal et al., 2005 [19]. As also found for the gel with *D. magna *fed with 100% *S. obliquus*, the four bands between 17 and 22 kDa were also assigned to the proteases 802 and 448, although the two bands between 17 and 19 kDa (chymotrypsin 802) of the gel with *D. magna *fed with 20% NIVA Cya 43 had another apparent molecular weight. Although no longer active in the SDS-PAGE (no hydrolytic activity), the chymotrypsin 802 at 18 kDa was still found at this position in the LC-MS/MS analysis. These three different bands from the two SDS-PAGEs of both homogenates were all assigned to CT802, and thus represent three different isoforms of the same protease. In the homogenate of *Daphnia *fed with 20% NIVA Cya 43, the bands between 21 and 22 kDa identified as protease 448 were the same as in the gel with homogenate of *Daphni*a grown on 100% *S. obliquus*. The protease at 20 kDa was identified as protease 383, another chymotrypsin. The proteases of both homogenates in the SDS-PAGE are accordingly assigned (Fig. 3).

### Expression of serine proteases after 24 h

**Figure 5:**
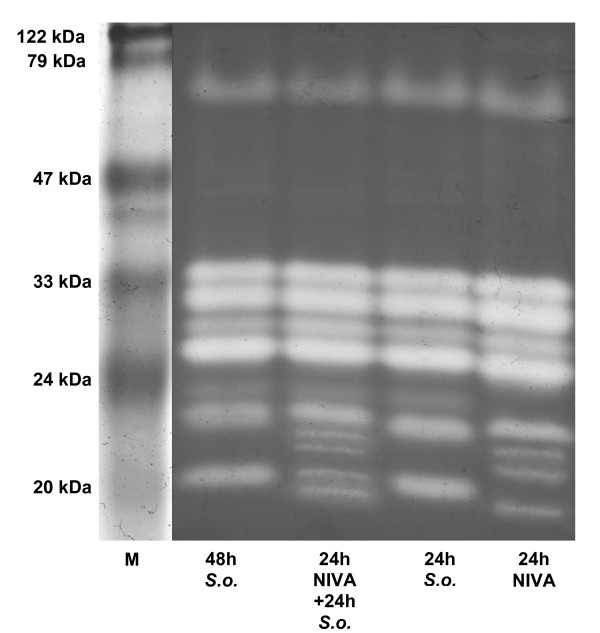
**SDS-PAGE of homogenate after 24 and 48 h food treatments**. Activity stained SDS-PAGE of homogenate from *D. magna *grown on 100% *S. obliquus *for 24 h ("24 h *S.o*.") or 48 h ("48 h *S.o*."), *D. magna *grown on 20% NIVA CYA 43 (with chymotrypsin inhibitors) and 80% *S. obliquus *for 24 h ("24 h NIVA") or for another 24 h on 100% *S. obliquus *("24 h NIVA + 24 h *S.o*."). White bands indicate active proteases. M = marker proteins.

The protease pattern of *D. magna *grown on 100% *S. obliquus *was the same after 24 as after 48 h (Fig. 5). When fed with 20% NIVA Cya 43, a shift in the protease pattern of the daphnids already took place after 24 h. A subsequent transfer of the animals to 100% *S. obliquus *for another 24 h led to an intermediate pattern with active proteases from both treatments, the 20% NIVA CYA 43 and the 100% *S. obliquus *treatment (Fig. 5).

**Figure 6:**
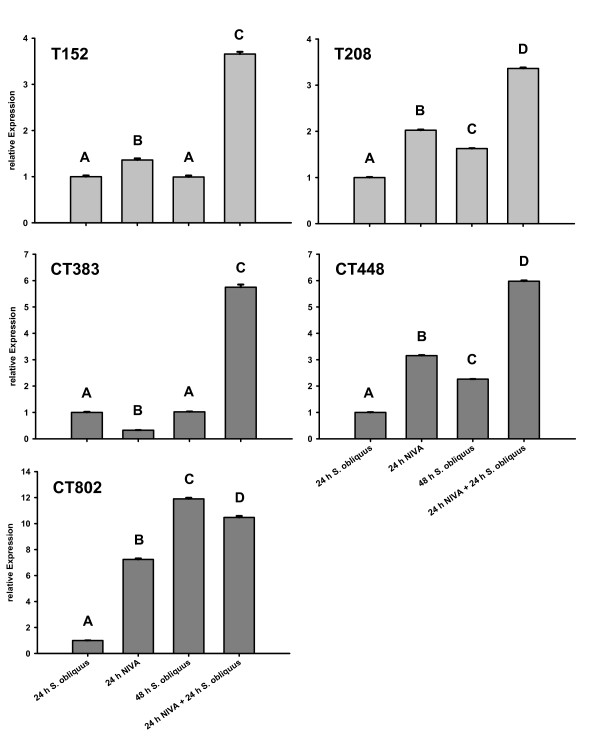
**relative gene expressions of proteases of *D. magna *grown on different food treatments after 24 and 48 h**. Relative gene expression of proteases form *D. magna *grown on 100% *S. obliquus *for 24 h or 48 h ("24 h *S.obliquus*" or "48 h *S.obliquus*"), or grown on 20% *M. aeruginosa *(with chymotrypsin inhibitors) NIVA CYA 43 and 80% *S. obliquus *for 24 h ("24 h NIVA"), or for another 24 h on 100% *S. obliquus *("24 h NIVA + 24 h *S.obliquus*"). Depicted is the mean relative expression (n = 3, ± SD) of trypsins (T152, T208) or chymotrypsins (CT448, CT383, CT802). Letters indicate a significant difference (p < 0.05) between calibrator (24 h *S. obliquus*) and cyanobacterial food treatments.

*Cyclophilin*, *SucDH *and *UBC *served as endogenous controls in the QPCR analysis. The relative expression of proteases had changed compared to the calibrator (24 h 100% *S. obliquus*; Fig. 6). All effects were significant (one way ANOVA: ***T152*: **F_3,8 _= 3745.9; p < 0.01; ***T208*: **F_3,8 _= 14892.5; p < 0.01; ***CT383*: **F_3,8 _= 6778; p < 0.01; ***CT448*: **F_3,8 _= 32554.5; p < 0.01; ***CT802*: **F_3,8 _= 10845.1 p < 0.01).

The proteases T152, T208 and CT448 showed consistent results. In the food treatment with 20% NIVA Cya 43 they were up-regulated after 24 h compared to the calibrator (24 h 100% *S. obliquus*; Fig. 6). *T208 *and *CT448 *were slightly up-regulated after 48 h on 100% *S. obliquus*, while for *T152 *the expression stayed the same as after 24 h on 100% *S. obliquus*; all three proteases showed the highest (3.4 to 5.97-fold) up-regulation in the treatment with 24 h 20% NIVA Cya 43/24 h *S. obliquus *compared to all other treatments.

In *CT383 *the results were the same except for the 24 h 20% NIVA Cya 43 treatment. Here, the expression was significantly lower than in the calibrator.

The regulation of *CT802*, however, differed considerably from that of the other proteases. Here, in the treatment with 24 h 20% NIVA Cya 43 the expression was up-regulated already over 7-fold compared to the calibrator. After 48 h *S. obliquus *the expression of *CT802 *increased 11.9-fold compared to 24 h *S. obliquus *and was therefore even more induced than in the treatment with 24 h 20% NIVA Cya 43. However, *CT802 *showed a higher level of induction after 24 h 20% NIVA Cya 43/24 h *S. obliquus *compared to the calibrator (10.5-fold), but was significantly lower than on 48 h *S. obliquus*.

### Expression of serine proteases in the presence of microcystin

**Figure 7:**
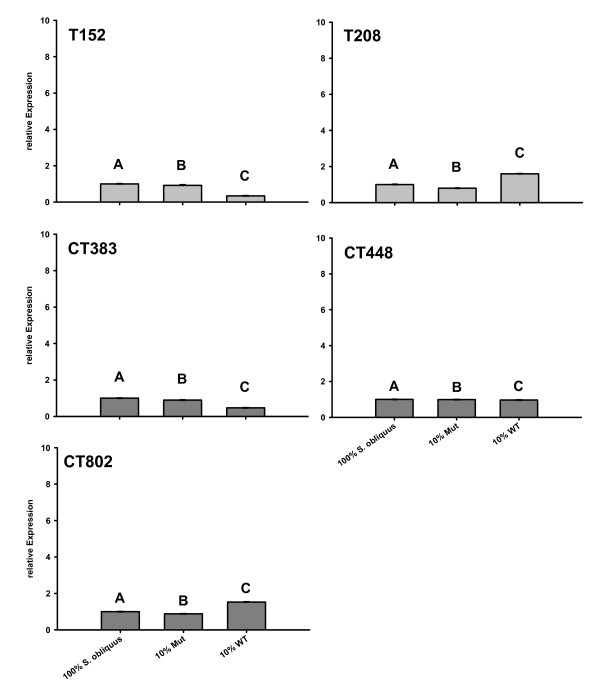
**relative expressions of proteases of *D. magna *grown in the presence or absence of microcystin**. *D. magna *were grown on three different food treatments (100% *S. obliquus*, 10% *M. aeruginosa *PCC 7806 Mut and 90% *S. obliquus*, or on 10% *M. aeruginosa *PCC 7806 WT (with microcystins) and 90% *S. obliquus*). Depicted is the mean relative expression (n = 3, ± SD) of trypsins (T152, T208) or chymotrypsins (CT448, CT383, CT802). Letters indicate a significant difference (p < 0.05) between calibrator (*S. obliquus*) and cyanobacterial food treatments. The y-axis is scaled to 10 to allow comparison with Fig. 4.

In order to test for effects of microcystin on protease expression, *D. magna *were fed with the microcystin-producing strain of PCC 7806 WT or with its mutant PCC 7806 Mut, which is incapable of producing microcystin. *Actin*, *SucDH *and *alpha-tubulin *served as endogenous controls in the QPCR analysis. There were significant (one way ANOVA: ***T152*: **F_2,6 _= 2071.3; p < 0.05; ***T208*: **F_2,6 _= 42016.5; p < 0.05; ***CT383*: **F_2,6 _= 36400.9; p < 0.05; ***CT448*: **F_2,6 _= 516; p < 0.05; ***CT802*: **F_2,6 _= 57697 p < 0.05) changes in relative expression between the treatments with 10% microcystin-free strain PCC 7806 Mut, 10% microcystin-containing PCC 7806 WT and 100% *S. obliquus *(Fig. 7). These changes in relative protease expression were low (0.34 to 1.6-fold) and negligible compared to the effects of the trypsin and chymotrypsin inhibitors from the 20% cyanobacterial food treatments with PCC 7806 Mut and NIVA Cya 43.

## Discussion

*Daphnia magna *fed with 100% green alga showed normal to high growth rates (Fig. 1), whereas the growth rate when fed with 20% cyanobacterial food was reduced. A reduction in growth rate or body length as an effect of cyanobacterial food has been observed previously in various *Daphnia *species: Growth reduction due to interference of filamentous cyanobacteria with the filtering apparatus [23,24] or due to a deficiency of polyunsaturated fatty acids (PUFAs) [25] or sterols [26], or because of the toxin content [27]. The *M. aeruginosa *strains used here were single-celled, small enough to be ingested, and did not contain microcystins [28,29]. Furthermore, a reduction in growth rate due to PUFA or sterol limitation can be excluded, since ≥ 80% of the dietary carbon was of eukaryotic origin, i.e. *S. obliquus *[30]. Therefore, in our case, growth rate reduction in response to cyanobacteria is probably caused by serine protease inhibitors, since the cyanobacterial strains used contain either mainly trypsin inhibitors (PCC 7806 Mut [19]) or strong chymotrypsin inhibitors (NIVA Cya 43 [17]).

The most important digestive serine proteases in *Daphnia magna *are trypsins and chymotrypsins [31]. For whole body homogenate, we found that the activity of chymotrypsins was ten times that of trypsins, corroborating findings for gut homogenate [31]. Von Elert et al. [31] observed nine protease bands in *D. magna *gut homogenate on an activity stained SDS-PAGE; the identical pattern is discernable for whole body homogenate (Fig. 2). This demonstrates that whole-body *Daphnia *homogenate can be used for further analyses, which is in accordance with the finding that whole-body activity shows only minor differences from the proteolytic activity of the gut associated activity [31]. Digestive proteases often have a compact molecular structure; this structure does not seem to be affected by SDS, as the protease pattern from native and SDS-PAGE was identical (Fig. 2). From another crustacean species, *Cancer pagurus*, it is known that trypsin and chymotrypsin activity is stable in regard to temperature and to many organic solvents [32]; our study shows that 2 M urea hardly affects trypsin and chymotrypsin activity in *Daphnia*, also indicating very stable proteases. Hence, it was not surprising that the proteases remained folded under denaturing conditions in SDS-PAGE, which resulted in a misinterpretation of the true molecular weight. A similar misinterpretation was made earlier for a trypsin from *Crangon *spp. [33]. In SDS-PAGE of *D. magna *homogenate, the proteases had apparent molecular weights between 17 and 75 kDa. Where possible, the true molecular weights of the proteases were calculated after translation of the cDNA sequences from the EST-database (http://www.nematodes.org/NeglectedGenomes/ARTHROPODA/Crustacea.html; [34]) from the start- to the stop-codon. The true molecular weights differed from the apparent molecular weights (Table 2).

**Table 2:** proteases of *D. magna*

Protease	Accession number	Primer forward	Primer reverse	Apparent MW [kDa]	True MW [kDa]
T152	DMC00580	5'-TGGATGCTCCATTGGACTTGA-3'	5'-CGGAAACGGTGACGATGATTC-3'	32-34	25.3
T208	DMC05983	5'-TGCGTTAGAGGAGTTGACGCT-3'	5'-TGAAGCTGACAACACCACGGT-3'	24-25	
CT383	DMC00886	5'-TTGGCACCTTCCACCGAAT-3'	5'-TCATCAGGACTGGAGAAACGC-3'	20	23.98
CT448	DMC01045	5'-TCATCAACGGAGCTGAGGCTA-3'	5'-AGAACCCACTGGTCGGAAATC-3'	21-22	26.4
T610	DMC00622			75	24.1
CT802	DMC00275	5'-TCAGATTGCCCAACCCCAT-3'	5'-TCCGCTGATGTGGAGCATATC-3'	17-19	24.7

Delineated are the names and the accession numbers of the proteases of *D. magna *from the EST-database search and the established forward and reverse primers for the QPCR analyses (no primers for T610 were established). Apparent molecular weights (MW) resulted from the comparison of the molecular weight marker with the visible protease bands in the SDS-PAGE, whereas true MW resulted from the translation of the sequences (start- to stop-codon) of the EST-database (http://www.nematodes.org/NeglectedGenomes/ARTHROPODA/Crustacea.html; [34]). No complete sequence was available for T208. (CT = chymotrypsin, T = trypsin).

The protease band pattern of *D. magna *grown on 20% PCC 7806 Mut was the same (Fig. 3) as on 100% *S. obliquus*. However, all trypsin bands showed reduced activity when the animals were fed with 20% PCC 7806 Mut, which is explicable by the release of cyanobacterial trypsin inhibitors from the *Microcystis *cells after ingestion by *Daphnia *during subsequent digestion in the gut. The activity of the chymotrypsins in the gel was not affected by this cyanobacterium, which is in accordance with the findings that PCC 7806 Mut mostly contains trypsin inhibitors [19]. A different effect was visible for *D. magna *fed with 20% NIVA Cya 43. This cyanobacterial strain affected both types of serine proteases. In spite of the high content of chymotrypsin inhibitors in this cyanobacterial strain [17], the chymotrypsin band at 21 kDa of the *Daphnia *homogenate slightly increased in intensity (Fig. 3). Although the protein was still detectable, the 18 kDa chymotrypsin was no longer active in the presence of dietary chymotrypsin inhibitors, leading to reduced total chymotrypsin activity in *D. magna *homogenate. This reduction of chymotrypsin activity was partly compensated for by the expression of the two new isoforms at 17 and 19 kDa. These two new isoforms proved to be active in the presence of chymotrypsin inhibitors (Fig. 3), indicating that these isoforms are less sensitive against the cyanobacterial inhibitors than the 18 kDa chymotrypsin. The additional expression of less sensitive chymotrypsins strongly suggests that this regulatory response to dietary chymotrypsin inhibitors is adaptive to *D. magna*.

Despite the expression of additional chymotrypsins, the overall activity of these proteases was visibly reduced in the presence of dietary chymotrypsin inhibitors (Fig. 3), since the intensities of the chymotrypsin protease bands were weaker than those of the chymotrypsin bands in the 100% *S. obliquus *treatment.

Moreover, chymotrypsin inhibitors led to a strong increase in trypsin activity. This can be considered to be an indirect effect of the chymotrypsin inhibitors and might be a compensatory physiological response of *D. magna *to cope with a reduced chymotrypsin activity.

In summary, the ingestion of the trypsin inhibitors cyanopeptolins [16,18,19] from PCC 7806 Mut only led to a decrease in trypsin activity of *D. magna*, whereas the chymotrypsin inhibitors nostopeptin BN920 and cyanopeptolin 954 [17] from NIVA Cya 43 not only affected chymotrypsin, but also led to an increase in trypsin activity as a putative compensatory response.

The previous classification of five protease bands on SDS-PAGE as trypsins was based on the inhibition by synthetic inhibitors, and the remaining active protease bands were merely postulated to be chymotrypsins [19]. Here, via LC-MS/MS and subsequent database search, all *Daphnia *proteases were clearly identified as trypsins or chymotrypsins and assigned to protease genes. The reason for two bands in the same lane in the SDS-PAGE referring to the same protease gene might be protein ripening or unspecific proteolytic degradation during the gel run. Alternatively, it might be attributed to the presence of splice variants or isoforms stemming from two different gene copies simultaneously active in *Daphnia*.

The two homogenates, which showed differences in the protease band pattern (100% *S. obliquus *and 20% NIVA Cya 43; Fig. 3) were analysed by LC-MS/MS (Appendices 1 and 2). The chymotrypsin bands, which were identified as CT802, showed a different molecular weight pattern in the two homogenates (Fig. 3). Although not visible as an active protease in the 20% NIVA Cya 43 treatment on the SDS-PAGE (Fig. 3), the CT802 protein at 18 kDa still was found. This result points to total inhibition of the 18 kDa isoform of CT802 due to dietary chymotrypsin inhibitors from NIVA Cya 43. However, other isoforms of CT802 which were more resistant to the inhibitor were expressed, and it is reasonable to assume that these isoforms stem from different gene copies. The *D. pulex *genome has been shown to contain a surprisingly high number of gene duplicates, leading to lineage-specific gene family expansions, which resulted in high numbers of genes (e.g. peptidases in *D. pulex *[35]). Rapid gene family expansions in phenotypically important genes suggest scenarios wherein adaptive natural selection favours additional copies, e.g. for adaptation to increased dosage [36]. In several insects, amplification of different esterase genes was the reason for resistance to organophosphate pesticides [37-39]; resistant strains of the mosquito *Culex pipiens *even showed a 250-fold increase in copy numbers [40].

To test if the observed changes in activity of the proteases on the SDS-PAGEs are caused by a change in gene expression, the relative expression of two trypsin genes (*T152, T208*) and of three chymotrypsin genes (*CT383, CT448, CT802*) in *D. magna *fed with three different food treatments were analysed with QPCR (Fig. 4). In the treatment with 20% NIVA Cya 43, both trypsins and *CT448 *were up-regulated by a factor of between 5 and 23. These results are reflected in the increased activity that was visible on the SDS-PAGE (Fig. 3). However, although the relative expression of *CT383 *and *CT802 *comparably increased (Fig. 4), only low activity of CT383 and of the two newly expressed isoforms of CT802 was observed on the SDS-PAGE (Fig. 3). One explanation for the low activity of CT802 might be that the new isoforms are not as active as CT802 in *D. magna *fed with green alga. CT383, of which no isoforms were detectable, might be more sensitive to the chymotrypsin inhibitors, so that the higher expression of *CT383 *might have been insufficient to compensate for simultaneous inhibition of the CT383 protein.

The 1.4 to 6-fold increase of the relative trypsin expression in *D. magna *fed with 20% PCC 7806 Mut was not visible as increased activity in the gel (Fig. 3); the same is true for the increase in chymotrypsin expression (2- to 3-fold; Fig. 3). SDS-PAGE is not sensitive enough to quantify protease activity, as doubling the amount of *Daphnia *homogenate in SDS-PAGE did not lead to an apparent increase in protease activity on the gels (data not shown). Hence, it is not surprising that the change in expression of trypsins and chymotrypsins of *Daphnia *from up to 5-fold was not visible as an increase in activity in SDS-PAGE.

Effects of different food treatments on the activity of digestive proteases in *D. magna *were already observed after 24 hours, which means that only a short period is required for *D. magna *to respond to the occurrence of dietary protease inhibitors. Such a rapid physiological response seems to be highly adaptive, since newborn *Daphnia *have to establish an optimal protease pattern quickly after birth to be able to initiate digestion. Gustafsson et al. [12] have shown that increased tolerance to microcystin-containing cyanobacteria was transferred to the offspring generations. If such a mechanism is also true for dietary protease inhibitors, maternal transfer might also lead to a fast establishment of an optimal protease pattern in newborn *Daphnia*.

Nevertheless, the additional expression of isoforms after 24 h and the concurrently observed up-regulation of protease expression of *Daphnia *fed with cyanobacteria are allocating additional resources to these proteins and can be assumed to be costly. To render the induction of proteases an evolutionary stable strategy, the level of expression should be tightly linked to the level of dietary protease inhibitors. However, such an immediate down-regulation of protease expression after sudden removal of dietary protease inhibitors was not observed, and an intermediate band pattern was visible in SDS-PAGE (Fig. 5). These observations can easily be explained by the fact that cyanobacterial mass developments in nutrient-rich lakes usually last for several months in summer so that the disappearance of cyanobacteria is a gradual process that can take several weeks [4]. Hence, the disappearance of cyanobacterial protease inhibitors will be considerably slower under field conditions than under our experimental conditions, which means that *Daphnia *are fully capable of adjusting the expression level of proteases to the presence of protease inhibitors in the natural diet.

As CT802 was the only protease showing a completely different response to cyanobacterial protease inhibitors in QPCR analyses, it has been concluded that *CT802 *must be regulated in a manner different from the other proteases. Interestingly, CT802 is the only protease which expresses new isoforms after *Daphnia *had been fed with dietary cyanobacterial food (Fig. 3 and 5). Further investigation of the regulation and of the processing leading to these new isoforms is needed. However, protease inhibitors are obviously a strong trigger for up-regulation of protease expression and for an induction of new isoforms. These protease inhibitors might also exert a strong selection pressure on *Daphnia *proteases themselves.

Numerous studies have focused on microcystins as the only reason for decreased fitness in *Daphnia *due to cyanobacteria, since microcystin LR is known to inhibit protein phosphatases of *Daphnia *in-vitro [41]. However, other secondary metabolites also have proved to have adverse effects on *Daphnia *[42,43]. The most widespread group of cyanobacterial secondary metabolites are protease inhibitors, which appear in nearly all cyanobacterial blooms [14,15], whereas this is not the case for microcystins. Von Elert et al. [31] have shown that the most important group of digestive enzymes in *Daphnia *are trypsins and chymotrypsins; these enzymes are indeed inhibited in vitro by specific cyanobacterial inhibitors [19]. By differentiating the effects of PCC 7806 WT and its microcystin-deficient mutant on *Daphnia*, both of which are known to contain strong trypsin inhibitors [19], the negative effects of microcystin-producing cyanobacteria on *Daphnia *could only be assigned to this compound to a limited degree [44,45]. QPCR results showed no difference in the regulation of proteases in the gut of *D. magna *between PCC 7806 WT and Mut, which clearly demonstrates that the interaction of cyanobacterial protease inhibitors with digestive proteases in *Daphnia *is not affected by microcystins. Protease inhibitors should affect gut proteases of *Daphnia *immediately after ingestion of the cyanobacterial food particles and the subsequent release of inhibitors during digestion, before microcystins come in contact with their targets, i.e. protein phosphatases I and II. This suggests that the tolerance of digestive proteases against dietary protease inhibitors in *Daphnia *coexisting with cyanobacteria should be under strong positive selection, even in the presence of other cyanobacterial inhibitors.

*Daphnia *serine proteases have been shown in-vitro to be inhibited by specific cyanobacterial inhibitors [19]. Here for the first time it was shown that a *D. magna *clone in-situ physiologically responds to dietary cyanobacterial protease inhibitors by phenotypic plasticity of the targets of these specific inhibitors, i.e. *Daphnia *gut proteases. The finding that *D. magna *responds to dietary protease inhibitors by up-regulation of protease expression on the RNA-level and by the expression of new and less-sensitive protease isoforms on the protein level strongly suggest that the observed phenotypic plasticity is adaptive.

## Conclusion

To our knowledge this is the first report on physiological plasticity in *D. magna *in response to the most widely spread cyanobacterial inhibitors, i.e., protease inhibitors. We have been able to show distinct physiological responses to dietary trypsin and chymotrypsin inhibitors. These physiological responses involve increased expression of the targets of these inhibitors, digestive trypsins and chymotrypsins, and the expression of less-sensitive isoforms. Clearly these regulatory responses are adaptive for *D. magna *as they increase the capacity for protein digestion in the presence of dietary protease inhibitors. It is therefore reasonable to assume that the kind and extent of these responses in protease expression determine the degree of growth rate reduction in *D. magna *in the presence of cyanobacteria with protease inhibitors. These physiological responses proved to be independent from microcystin effects, as there only were negligible differences between protease expression of *D. magna *fed with *M. aeruginosa *PCC7806 WT and its microcystin-free mutant. *Daphnia *neonates respond very quickly to cyanobacterial food (24 h), which supports the assumption that dietary cyanobacterial protease inhibitors exert a strong selection pressure on *Daphnia *proteases themselves.

## Methods

### Cultivation of *Daphnia magna*

The *Daphnia magna *clone 'Binnensee' [46] was cultivated in 1 l filtered (0.2 μm) water from a nearby pond (Aachener Weiher in Cologne) and fed daily with a saturating concentration of *Scenedesmus obliquus *SAG 276-3a. The water and the food were exchanged every two days. Neonates from the 3^rd ^clutch which were no more than twelve hours old were used for the experiments.

### Cultivation of algae and cyanobacteria

*Scenedesmus obliquus *SAG 276-3a was grown in sterile 5 l semi-continuous batch cultures on cyanophycea-medium [47] at 20°C and constant light (150 μE). Every day 1 l of algal suspension was exchanged with fresh medium.

The cyanobacteria *Microcystis aeruginosa *NIVA Cya 43, a microcystin-free strain [28], the wild-type *M. aeruginosa *PCC 7806 WT and its mutant *M. aeruginosa *PCC 7806 Mut [29] (further referred to as NIVA Cya 43, PCC 7806 WT and PCC 7806 Mut) were cultivated in chemostats on cyanophycea-medium at 20°C and constant light (50 μE). The dilution rate was 0.23 d^-1^.

### Somatic growth on different food treatments

Growth experiments were performed in 250 ml of 0.2 μm filtered pond water for six days with five neonates per replicate and a food concentration of 2 mg C/l. The treatments were either 100% *Scenedesmus obliquus*, 20% NIVA Cya 43 and 80% *S. obliquus *or 20% PCC 7806 Mut and 80% *S. obliquus*. Each treatment was run in triplicate. Water and food were exchanged daily. The dry weight of the animals was used to calculate the somatic growth rate (g, d^-1^) of each treatment according to Wacker et al., 2001 [48] using the formula g = (ln x_te _- ln x_tb_)/Δt, for which x_te _is the weight after six days, x_tb _is the weight at the start of the experiment and Δt is the test duration, i.e. six days.

### Preparation of *Daphnia *and gut homogenates

Neonates of *Daphnia magna *grown on 2 mg C/l of *S. obliquus *for six days were transferred to 5 μl 2 mM Dithiothreitol (DTT) per animal and were homogenized with a pestle. The homogenate was centrifuged for 3 min at 14,000× g. The protein concentration of the supernatant - the *Daphnia*-homogenate - was analyzed using a Qubit fluorometer and the appropriate Quant-iT™ Protein Assay Kit (Invitrogen, Paisley, UK) as according to the manufacturer's advice.

Guts including the hepatopancreases of *D. magna *grown on 2 mg C/l of *S. obliquus *for six days were separated and transferred to 5 μl 2 mM DTT per gut, as according to Agrawal et al., 2005 [19] and treated in the same way as the *Daphnia*-homogenate.

### Activity and stability of serine proteases of *D. magna*

Chymotrypsin activity of the *Daphnia*-homogenate was measured photometrically using the artificial substrate N-Succinyl-Alanine-Alanine-Proline-Phenylalanine-*para*-Nitroanilide (S(Ala)_2_ProPhepNA; Sigma, Munich, Germany; [31]). 10 μl *Daphnia*-homogenate was mixed with 980 μl 0.1 M potassium-phosphate-buffer, pH 6.5. The buffer contained 125 μM S(Ala)_2_ProPhepNA and 1% Dimethyl sulfoxide (DMSO; Sigma, Munich, Germany). The change in absorption was measured at a wavelength of 390 nm at 30°C continuously over 10 min. The trypsin activity was measured using the artificial substrate N-Benzoyl-Arginine-*para*-Nitroanilide (BApNA; Sigma, Munich, Germany; [31]). 10 μl *Daphnia*-homogenate was mixed with 895 μl 0.1 M potassium-phosphate-buffer, pH 6.5. The buffer contained 1.88 mM BApNA and 7.5% DMSO. The change in absorption was measured at a wavelength of 390 nm at 30°C continuously over 10 min. To test the stability of proteases, the buffers with *Daphnia*-homogenate were incubated for 2 min with 2 M urea before the kinetic analysis; activity was compared to the control without urea. Protein concentrations were analysed with the Qubit fluorometer.

### SDS-PAGE and native gel of *Daphnia*-homogenate

*Daphnia*-homogenate (20 μg protein) with 5 μl 4× Laemmli-buffer [49] was loaded on a 12% SDS-polyacrylamide gel and run at 200 V at 6°C. *Daphnia*-homogenate (20 μg protein) with 5 μl 4× Laemmli-buffer without SDS was loaded on a native (no SDS) 12% polyacrylamide gel and run at 200 V at 6°C with SDS-free running buffer. After the run the gels were activity stained according to Von Elert et al., 2004 [31]; the gels were washed and incubated with agitation for 30 min at 4°C and for another 90 min at 20°C in 50 mM Tris-HCl (pH 9) containing 0.75% (w/v) casein Hammerstein. During this time the proteases, which were released from SDS, refolded and digested the casein. Gels were washed, fixed in 12% Trichloracetic acid, stained with 0.25% Coomassie Brilliant Blue in methanol:acetic acid:water (50:10:40, by vol.) and destained in methanol:acetic acid:water (42:8:50, by vol.). The molecular weights of the visible proteases were compared between the two methods. The marker on all PAGEs was the peqGold Prestained Protein Marker III (peqlab, Erlangen, Germany).

### LC/MS-MS analysis of protease bands

Proteases were subjected to purification prior to LC-/MSMS analysis as follows:

200 live *D. magna *grown on 100% *S. obliquus *or 20% *M. aeruginosa *and 80% *S. obliquus *were homogenized and centrifuged as described above. 500 μl of the supernatant were mixed with 500 μl ultrapure water and were loaded onto a strong anion-exchanger column (SAX; Varian, No. 1210-2044; Palo Alto, CA, USA). The proteases bound to the column were eluted with one bed volume of 0.9 M NaCl and dialysed at 4°C for 24 h in 1 l of 10 mM imidazole-buffer, pH 6.9. Subsequently the proteases were precipitated with ice-cold 70% acetone. The pellet (centrifugation: 10 min, 14,000× g at 4°C) was lyophilized and resuspended in 40 μl of ultrapure water. It was mixed with 10 μl 4× Laemmli-buffer, loaded onto a 12% SDS-PAGE and Coomassie-stained after electrophoresis.

Coomassie-stained protein bands were excised from the gel, chopped into cubes and washed three times with acetonitrile-water (1:1). The gel pieces were shrunk with neat acetonitrile, allowed to rehydrate in 50 mM NH_4_HCO_3 _and dried in a speedvac. 10 mM DTT in 50 mM NH_4_HCO_3 _were added to the dried gel pieces, and proteins were reduced for 45 min at 56°C. To alkylate reduced cysteine residues, the remaining liquid was removed, and an equal volume of 50 mM iodoacetamide in 50 mM NH_4_HCO_3 _was added. The reaction was allowed to proceed for 30 min in the dark. Prior to in-gel digestion, the gel pieces were washed and dried as above. The gel pieces were allowed to rehydrate in an ice-cold solution of 12.5 ng/μl semiTrypsin semi-trypsin (for homogenate of *Daphnia *fed with 20% NIVA Cya 43) or Trypsin (for 100% *S. obliquus *homogenate; sequencing grade, Promega) in 10 mM NH_4_HCO_3_. After 45 min on ice, excessive enzyme solution was replaced by 5 - 20 μl of buffer without enzyme, and proteins were digested at 37°C overnight. The digestion was stopped by the addition of 5 - 20 μl 1% TFA, and peptides were extracted for 30 min at 37°C.

LC-MS/MS data for the 100% *S. obliquus *homogenate were acquired according to Hanisch et al., 2009 [50]. For the homogenate of *Daphnia *fed with 20% NIVA Cya 43, LC-MS/MS data were acquired on a HCT ETD II iontrap mass spectrometer (Bruker Daltonics, Bremen, Germany) equipped with a nano ESI source (Bruker Daltonics, Bremen, Germany). Samples were introduced by an easy nano LC system (Proxeon, Odense, Denmark) using a vented column setup comprising a 0.1-mm-by-20-mm trapping column and a 0.075-by-100-mm analytical column, both self packed with ReproSil-Pur C18-AQ, 5 μm (Dr. Maisch, Ammerbuch, Germany). 5 μl to 18 μl of sample were aspirated into the sample loop, and a total of 25 μl was loaded onto the trap column at a flow rate of 6 μl/min. Loading pump buffer was 0.1% formic acid (FA). Peptides were eluted with a gradient of 0% to 35% acetonitrile (ACN) in 0.1% FA over 20 min and a column flow rate of 300 nl/min. Subsequently the ACN content was raised to 100% over 2 min, and the column was regenerated in 100% ACN for additional 8 min.

Data-dependent acquisition of MS and tandem MS (MS/MS) spectra was controlled by the Compass 3.0 software. MS1 scans were acquired in standard enhanced mode. Five single scans in the mass range from *m/z *400 to *m/z *1400 were combined for one survey scan. Up to three doubly and triply charged ions rising above a given threshold were selected for MS/MS experiments. Ultrascan mode was used for the acquisition of MS2 scans in the mass range from m/z 100 m/z 1600, and three single scans were added up. The ion charge control value was set to 250000 for all scan types. Peaklists in mascot generic format (mgf) were generated from the raw data by using the Data Analysis software module (Bruker Daltonics, Bremen, Germany).

Proteins were identified by using a local installation of MASCOT 2.2 (Matrix Science Ltd, London, UK). All serine proteases (13) from a *D. magna *EST-database (http://www.nematodes.org/NeglectedGenomes/ARTHROPODA/Crustacea.html; [34]) and a complete *D. pulex *database (http://wfleabase.org/; [20]; release: July 2007) were used. The database search could be reduced to serine proteases because Agrawal et al. [19] assigned all visible protease bands in SDS-PAGE to serine proteases. Searches were submitted via Proteinscape 2.0 (Bruker Daltonics, Bremen, Germany) with the following parameter settings: enzyme "semitrypsin", fixed modifications "carbamidomethyl", optional modifications "Methionine oxidation" and missed cleavages "1". The mass tolerance was set to 0.4 Da for peptide and fragment spectra. The most probable hits for the bands of the SDS-PAGEs were determined by the number of matched peptides, the percent-wise sequence coverage, and the probability MOWSE score.

### Food treatments: SDS-PAGE and quantitative real-time PCR (QPCR)

Fifteen neonates of *D. magna *clone Binnensee were grown on 2 mg C/l in 1 l filtered pond water on either 100% *S. obliquus*, 20% NIVA Cya 43 and 80% *S. obliquus *or on 20% PCC 7806 Mut and 80% *S. obliquus*. Each treatment was run in triplicate. The water and the food were exchanged daily. The experiment was stopped after six days, after which half of the animals were used for 12% SDS-PAGE followed by activity staining; RNA was extracted from the other half using the RNeasy Mini Kit (Qiagen, Hilden, Germany) following the manufacturer's instructions. RNA was purified with DNase I (Fermentas, St. Leon-Rot, Germany) and reverse transcribed with High-capacity cDNA Reverse Transcription Kit with RNase Inhibitor (Applied Biosystems, Foster City, CA, USA).

Nine different housekeeping genes recently introduced for QPCR in *D. magna *by Heckmann et al., 2006 [51] were used in QPCR analysis: *actin, alpha-tubulin, cyclophilin, glyceraldehyde-3-phosphate dehydrogenase (GapDH), succinate dehydrogenase (SucDH), TATA-box binding protein (TBP), ubiquitin conjugating enzyme (UBC), 18S ribosomal RNA (18S), and 28S ribosomal RNA (28S)*. A normalisation factor was calculated based on the endogenous controls assessed by geNorm [52] according to Schwarzenberger et al., 2009 [53].

Forward and reverse primers for QPCR were established from the EST-database for five proteases that were found in the SDS-PAGEs (http://www.nematodes.org/NeglectedGenomes/ARTHROPODA/Crustacea.html[34]; Table 2). Real Time PCR with different concentrations of cDNA from six-day-old *D. magna *grown on *S. obliquus *was performed, and amplification efficiencies for the protease primers were calculated as according to Livak et al., 2001 [54] using the formula AE = 10^(-1/slope), where AE is the amplification efficiency. qRT- PCR was performed as according to Schwarzenberger et al., 2009 [53]. *D. magna *fed with 100% *S. obliquus *served as calibrator, which was always set as 1.

### Microcystin effect on the expression of serine proteases

To investigate the effect of microcystin on *Daphnia *gut proteases, 15 neonates of *Daphnia magna *Binnensee were grown on 2 mg C/l in 1 l filtered pond water on either 100% *S. obliquus*, 10% PCC 7806 WT and 90% *S. obliquus *or on 10% PCC 7806 Mut and 90% *S. obliquus *for six days. Since the mortality after this time on 20% of the microcystin-containing WT was too high, *D. magna *were grown in the presence of 10% of either cyanobacterial strain. Each treatment was run in triplicate. The medium was exchanged daily. QPCR was conducted following Schwarzenberger et al., 2009 [53].

### Expression of serine proteases after 24 h

Fifteen neonates of *Daphnia magna *Binnensee were grown on 2 mg C/l in 1 l filtered pond water on either 100% *S. obliquus *or on 20% NIVA Cya 43 and 80% *S. obliquus*. Each treatment was run in triplicate. RNA and proteins were extracted from half of the animals after 24 hours. Thereafter the remaining animals grown on the mixture with cyanobacteria and the animals grown on the green alga were further cultivated for another 24 hours on 100% *S. obliquus*. Again proteins and RNA were extracted, and Real Time PCR was conducted. The proteases were activity stained after SDS-PAGE.

### Statistics

The statistics were conducted with the program Statistica 6.0 (StatSoft, Inc., Tulsa, OK, USA). The data were analysed via one-way ANOVA and a post-hoc analysis (Tukey HSD). A Levene's Test was conducted to ensure homogenous variances. The data were ln (x+1) transformed when needed. The level of significance was p < 0.05.

## Authors' contributions

EVE and AS designed and coordinated the study. AS performed all practical aspects under the supervision of EVE except for the LC-MS/MS analysis which was performed by SM. AZ and PK provided advice to parts of the project. The work was supported by a grant to EVE (DFG EL 179/6-1). All authors contributed to, read, and approved the final manuscript.

## Supplementary Material

Additional file 1 **Results of LC-MS/MS analysis of *Daphnia *homogenate**. *D. magna *were raised on 100% *S. obliquus*. Depicted are the results of LC-MS/MS analysis (apparent molecular weight of the cut band, number of the matched peptides, sequence of the matched peptides, sequence coverage with the hits in the database, probability based mowse score, hit in the database and the function of the hits). Click here for file

Additional file 2 **Results of LC-MS/MS analysis of homogenate of *D. magna *grown on 20% *M. aeruginosa *NIVA CYA 43**. *D. magna *were raised on 80% *S. obliquus *and 20%*M.aeruginosa *NIVA CYA 43. Depicted are the results of LC-MS/MS analysis (apparent molecular weight of the cut band, number of the matched peptides, sequence of the matched peptides, sequence coverage with the hits in the database, probability based mowse score, hit in the database and the function of the hits). Click here for file
