# Supplementary material for: Gene expression and activity of digestive proteases in Daphnia: effects of cyanobacterial protease inhibitors
Source: BMC Physiol. 2010 May 4;10:6. doi: 10.1186/1472-6793-10-6 (PMC2873468; doi:10.1186/1472-6793-10-6)
Supplement: Additional file 1 — Results of LC-MS/MS analysis of Daphnia homogenate. D. magna were raised on 100% S. obliquus. Depicted are the results of LC-MS/MS analysis (apparent molecular weight of the cut band, number of the matched peptides, sequence of the matched peptides, sequence coverage with the hits in the database, probability based mowse score, hit in the database and the function of the hits). [file 1472-6793-10-6-S1.DOC]

**Appendix 1 – results of LC-MS/MS analysis of *Daphnia* homogenate**

*D. magna* were raised on 100% *S. obliquus*. Depicted are the results of LC-MS/MS analysis (apparent molecular weight of the cut band, number of the matched peptides, sequence of the matched peptides, sequence coverage with the hits in the database, probability based mowse score, hit in the database and the function of the hits).

| Band (apparent  MW) | number of  matched peptides | sequence  of matched peptide | sequence coverage | Probability based mowse score | hit | function |
| --- | --- | --- | --- | --- | --- | --- |
| 18 kDa | 3 | LDAADEPTR | 9% | 143 | 802 | chymotrypsin |
|  |  | IINDVALIR |  |  |  |  |
|  |  | LDAADEPTRVEVR |  |  |  |  |
| 21 kDa | 2 | ITETERLEIR | 10% | 105 | 448 | chymotrypsin |
|  |  | TADGPGGISPTLQK |  |  |  |  |
|  | 1 | TADGPGGISPTLQK |  | 86 | [WFes0109692](http://134.95.232.242/mascot/cgi/protein_view.pl?file=../data/20081010/F050511.dat&hit=WFes0109692&px=1&protscore=86.05) | n.n. |
| 22 kDa | 2 | ITETERLEIR | 10% | 66 | 448 | chymotrypsin |
|  |  | TADGPGGISPTLQK |  |  |  |  |
| 24 kDa | 2 | VVAGEHSLR | 7% | 109 | 152/ 208 | trypsin |
|  |  | TDSGLEQNR |  |  |  |  |
|  | 2 | VVAGEHSLR |  | 109 | [WFes0171720](http://134.95.232.242/mascot/cgi/protein_view.pl?file=../data/20081010/F050509.dat&hit=WFes0171720&px=1&protscore=108.87) | n.n. |
|  |  | TDSGLEQNR |  |  |  |  |
|  | 1 | AIFVDGGIHAR |  | 56 | [WFes0141987](http://134.95.232.242/mascot/cgi/protein_view.pl?file=../data/20081010/F050509.dat&hit=WFes0141987&px=1&protscore=56.39) | n.n. |
| 26 kDa | 2 | VVAGEHSLR | 7% | 114 | 152/ 208 | trypsin |
|  |  | TDSGLEQNR |  |  |  |  |
|  | 2 | VVAGEHSLR |  | 114 | [WFes0171720](http://134.95.232.242/mascot/cgi/protein_view.pl?file=../data/20081010/F050508.dat&hit=WFes0171720&px=1&protscore=114.24) | n.n. |
|  |  | TDSGLEQNR |  |  |  |  |
| 32 kDa | 2 | VVAGEHSLR | 7% | 79 | 152/ 208 | trypsin |
|  |  | TDSGLEQNR |  |  |  |  |
|  | 2 | VVAGEHSLR |  | 79 | [WFes0171720](http://134.95.232.242/mascot/cgi/protein_view.pl?file=../data/20081010/F050507.dat&hit=WFes0171720&px=1&protscore=79.14) | n.n. |
|  |  | TDSGLEQNR |  |  |  |  |
| 34 kDa | 2 | VVAGEHSLR | 7% | 122 | 152/ 208 | trypsin |
|  |  | TDSGLEQNR |  |  |  |  |
|  | 2 | VVAGEHSLR |  | 122 | [WFes0171720](http://134.95.232.242/mascot/cgi/protein_view.pl?file=../data/20081010/F050506.dat&hit=WFes0171720&px=1&protscore=121.55) | n.n. |
|  |  | TDSGLEQNR |  |  |  |  |
|  | 1 | AIVVDGGIHAR |  | 48 | [WFes0128520](http://134.95.232.242/mascot/cgi/protein_view.pl?file=../data/20081010/F050506.dat&hit=WFes0128520&px=1&protscore=48.36) | n.n. |
