# Supplementary material for: Gene expression and activity of digestive proteases in Daphnia: effects of cyanobacterial protease inhibitors
Source: BMC Physiol. 2010 May 4;10:6. doi: 10.1186/1472-6793-10-6 (PMC2873468; doi:10.1186/1472-6793-10-6)
Supplement: Additional file 2 — Results of LC-MS/MS analysis of homogenate of D. magna grown on 20% M. aeruginosa NIVA CYA 43. D. magna were raised on 80% S. obliquus and 20%M.aeruginosa NIVA CYA 43. Depicted are the results of LC-MS/MS analysis (apparent molecular weight of the cut band, number of the matched peptides, sequence of the matched peptides, sequence coverage with the hits in the database, probability based mowse score, hit in the database and the function of the hits). [file 1472-6793-10-6-S2.DOC]

**Appendix 2 – results of LC-MS/MS analysis of homogenate of *D. magna* grown on 20% *M. aeruginosa* NIVA CYA 43**

*D. magna* were raised on 80% *S. obliquus* and 20% *M.aeruginosa* NIVA CYA 43. Depicted are the results of LC-MS/MS analysis (apparent molecular weight of the cut band, number of the matched peptides, sequence of the matched peptides, sequence coverage with the hits in the database, probability based mowse score, hit in the database and the function of the hits).

| Band  (apparent  MW) | number of  matched peptides | sequence of matched peptide | sequence coverage | Probability based mowse score | hit | function |
| --- | --- | --- | --- | --- | --- | --- |
| 75 kDa | 6 | LSSPLSLNTK | 22% | 33 | [610](../../../../D:%5CDokumente%20und%20Einstellungen%5C2%5CEigene%20Dateien%5CDoktorarbeit%20Anke%5CErgebnisse%5CProteomics%5Cgeklappt%5CNIVA%20B%5C7958_7969%5C7958%20VE%5Czmmk-mascot%5Cmascot%5Ccgi%5Cprotein_view9802.html) | trypsin |
|  |  | TEHEAYSSR |  | [39] |  |  |
|  |  | TEHEAYSSR |  | 46 |  |  |
|  |  | SIDSGDEQYSDILSK |  | 105 |  |  |
|  |  | IVGGTQASPNEFPYQISLR |  | [54] |  |  |
|  |  | IVGGTQASPNEFPYQISLR |  | 84 |  |  |
|  | 2 | ISDTLR | 6% | 31 | [152](../../../../D:%5CDokumente%20und%20Einstellungen%5C2%5CEigene%20Dateien%5CDoktorarbeit%20Anke%5CErgebnisse%5CProteomics%5Cgeklappt%5CNIVA%20B%5C7958_7969%5C7958%20VE%5Czmmk-mascot%5Cmascot%5Ccgi%5Cprotein_viewa299.html) | trypsin |
|  |  | TDSGLEQNR |  | 34 |  |  |
| 34 kDa | 20 | AGEHSLR | 45% | 68 | [152](../../../../D:%5CDokumente%20und%20Einstellungen%5C2%5CEigene%20Dateien%5CDoktorarbeit%20Anke%5CErgebnisse%5CProteomics%5Cgeklappt%5CNIVA%20B%5C7958_7969%5C7959%20VE%5Czmmk-mascot%5Cmascot%5Ccgi%5Cprotein_viewb50a.html) | trypsin |
|  |  | VVAGEHSL |  | 40 |  |  |
|  |  | GVDASILR |  | 54 |  |  |
|  |  | VAGEHSLR |  | 66 |  |  |
|  |  | VVAGEHSLR |  | 88 |  |  |
|  |  | IVGGTTVEPN |  | 36 |  |  |
|  |  | LDWIAANRG |  | 42 |  |  |
|  |  | TDSGLEQNR |  | 55 |  |  |
|  |  | AYGGTAANPEVYPS |  | 35 |  |  |
|  |  | AYGGTAANPEVYPSML |  | 48 |  |  |
|  |  | SGWGTTSSGGVISDTLR |  | [68] |  |  |
|  |  | SGWGTTSSGGVISDTLR |  | 86 |  |  |
|  |  | VSGWGTTSSGGVISDTLR |  | 104 |  |  |
|  |  | VVAGEHSLRTDSGLEQNR |  | 30 |  |  |
|  |  | IVGGTTVEPNSLPFQISLQ |  | 79 |  |  |
|  |  | IVGGTTVEPNSLPFQISLQ |  | [29] |  |  |
|  |  | IVGGTTVEPNSLPFQISLQR |  | 94 |  |  |
|  |  | GVASIIIHEDYRPLTFEND |  | 49 |  |  |
|  |  | GVASIIIHEDYRPLTFENDISL |  | 22 |  |  |
|  |  | GVASIIIHEDYRPLTFENDISLL |  | 45 |  |  |
|  | 3 | VSGLEQNR | 7% | 58 | [79](../../../../D:%5CDokumente%20und%20Einstellungen%5C2%5CEigene%20Dateien%5CDoktorarbeit%20Anke%5CErgebnisse%5CProteomics%5Cgeklappt%5CNIVA%20B%5C7958_7969%5C7959%20VE%5Czmmk-mascot%5Cmascot%5Ccgi%5Cprotein_view41bb.html) | trypsin |
|  |  | VVAGEHDLS |  | 22 |  |  |
|  |  | VVAGEHDLSQVSGLEQNR |  | 57 |  |  |
|  | 1 | TADGPGGISPTLQK | 5% | 73 | [448](../../../../D:%5CDokumente%20und%20Einstellungen%5C2%5CEigene%20Dateien%5CDoktorarbeit%20Anke%5CErgebnisse%5CProteomics%5Cgeklappt%5CNIVA%20B%5C7958_7969%5C7959%20VE%5Czmmk-mascot%5Cmascot%5Ccgi%5Cprotein_view96bc.html) | chymotrypsin |
| 32 kDa | 22 | AGEHSLR | 42% | [27] | [152](../../../../D:%5CDokumente%20und%20Einstellungen%5C2%5CEigene%20Dateien%5CDoktorarbeit%20Anke%5CErgebnisse%5CProteomics%5Cgeklappt%5CNIVA%20B%5C7958_7969%5C7960%20VE%5Czmmk-mascot%5Cmascot%5Ccgi%5Cprotein_view254f.html) | trypsin |
|  |  | AGEHSLR |  | 64 |  |  |
|  |  | AGEHSLR |  | [28] |  |  |
|  |  | VVAGEHSL |  | 37 |  |  |
|  |  | GVDASILR |  | 56 |  |  |
|  |  | VAGEHSLR |  | 62 |  |  |
|  |  | LDWIAANR |  | 51 |  |  |
|  |  | VVAGEHSLR |  | 67 |  |  |
|  |  | IVGGTTVEPN |  | 26 |  |  |
|  |  | LDWIAANRG |  | 43 |  |  |
|  |  | TDSGLEQNR |  | 69 |  |  |
|  |  | RVVAGEHSLR |  | 25 |  |  |
|  |  | AYGGTAANPEVYPS |  | 53 |  |  |
|  |  | SFFLDWIAANRG |  | 60 |  |  |
|  |  | SVDVPVVDDDTCNR |  | 22 |  |  |
|  |  | GWGTTSSGGVISDTLR |  | 97 |  |  |
|  |  | IVGGTTVEPNSLPFQI |  | 46 |  |  |
|  |  | SGWGTTSSGGVISDTLR |  | 101 |  |  |
|  |  | SGWGTTSSGGVISDTLR |  | [61] |  |  |
|  |  | GEHSLRTDSGLEQNR |  | 30 |  |  |
|  |  | VVAGEHSLRTDSGLEQNR |  | 49 |  |  |
|  |  | IVGGTTVEPNSLPFQISLQR |  | 72 |  |  |
|  | 12 | AGEHSLR | 18% | [27] | [208](../../../../D:%5CDokumente%20und%20Einstellungen%5C2%5CEigene%20Dateien%5CDoktorarbeit%20Anke%5CErgebnisse%5CProteomics%5Cgeklappt%5CNIVA%20B%5C7958_7969%5C7960%20VE%5Czmmk-mascot%5Cmascot%5Ccgi%5Cprotein_viewca1b.html) | trypsin |
|  |  | AGEHSLR |  | 64 |  |  |
|  |  | AGEHSLR |  | [28] |  |  |
|  |  | VVAGEHSL |  | 37 |  |  |
|  |  | GVDATILR |  | 50 |  |  |
|  |  | VAGEHSLR |  | 62 |  |  |
|  |  | VVAGEHSLR |  | 67 |  |  |
|  |  | TDSGLEQNR |  | 69 |  |  |
|  |  | RVVAGEHSLR |  | 25 |  |  |
|  |  | GEHSLRTDSGLEQNR |  | 30 |  |  |
|  |  | VVAGEHSLRTDSGLEQNR |  | 49 |  |  |
|  |  | IVGGTVVEPNSLPFQISLQR |  | 45 |  |  |
|  | 6 | LSVPSAK | 21% | 22 | [79](../../../../D:%5CDokumente%20und%20Einstellungen%5C2%5CEigene%20Dateien%5CDoktorarbeit%20Anke%5CErgebnisse%5CProteomics%5Cgeklappt%5CNIVA%20B%5C7958_7969%5C7960%20VE%5Czmmk-mascot%5Cmascot%5Ccgi%5Cprotein_view0e57.html) | trypsin |
|  |  | VSGLEQNR |  | 58 |  |  |
|  |  | VVAGEHDLS |  | 25 |  |  |
|  |  | LDWIAANR |  | 51 |  |  |
|  |  | IVGGTEVVPN |  | 35 |  |  |
|  |  | IVGGTEVVPNSLPFQISLQR |  | 69 |  |  |
|  | 3 | TADGPGGISPTLQK | 13% | 107 | [448](../../../../D:%5CDokumente%20und%20Einstellungen%5C2%5CEigene%20Dateien%5CDoktorarbeit%20Anke%5CErgebnisse%5CProteomics%5Cgeklappt%5CNIVA%20B%5C7958_7969%5C7960%20VE%5Czmmk-mascot%5Cmascot%5Ccgi%5Cprotein_viewabb6.html) | chymotrypsin |
|  |  | YIHPDWNPNTLTGDVAL |  | 32 |  |  |
|  |  | YIHPDWNPNTLTGDVALIK |  | 42 |  |  |
|  | 5 | GANIDNLR | 37% | 47 | [239](../../../../D:%5CDokumente%20und%20Einstellungen%5C2%5CEigene%20Dateien%5CDoktorarbeit%20Anke%5CErgebnisse%5CProteomics%5Cgeklappt%5CNIVA%20B%5C7958_7969%5C7960%20VE%5Czmmk-mascot%5Cmascot%5Ccgi%5Cprotein_view35e1.html) | serinprotease |
|  |  | IVGGVEVVPN |  | 38 |  |  |
|  |  | SLPFQVSLQR |  | 50 |  |  |
|  |  | DAAHCIAGANIDNLR |  | 37 |  |  |
|  |  | DAAHCIAGANIDNLR |  | [27] |  |  |
|  | 2 | LDAADEPTR | 5% | 53 | [802](../../../../D:%5CDokumente%20und%20Einstellungen%5C2%5CEigene%20Dateien%5CDoktorarbeit%20Anke%5CErgebnisse%5CProteomics%5Cgeklappt%5CNIVA%20B%5C7958_7969%5C7960%20VE%5Czmmk-mascot%5Cmascot%5Ccgi%5Cprotein_viewe512.html) | chymotrypsin |
|  |  | LDAADEPTRVEVR |  | 31 |  |  |
|  | 1 | ITLGAHDR | 3% | 26 | [383](../../../../D:%5CDokumente%20und%20Einstellungen%5C2%5CEigene%20Dateien%5CDoktorarbeit%20Anke%5CErgebnisse%5CProteomics%5Cgeklappt%5CNIVA%20B%5C7958_7969%5C7960%20VE%5Czmmk-mascot%5Cmascot%5Ccgi%5Cprotein_viewdb19.html) | chymotrypsin |
| 26 kDa | 19 | AGEHSLR | 39% | 64 | [208](../../../../D:%5CDokumente%20und%20Einstellungen%5C2%5CEigene%20Dateien%5CDoktorarbeit%20Anke%5CErgebnisse%5CProteomics%5Cgeklappt%5CNIVA%20B%5C7958_7969%5C7961%20VE%5Czmmk-mascot%5Cmascot%5Ccgi%5Cprotein_viewf159.html) | trypsin |
|  |  | GVDATILR |  | 56 |  |  |
|  |  | VVAGEHSLR |  | 75 |  |  |
|  |  | VVAGEHSLR |  | [70] |  |  |
|  |  | IVGGTVVEPN |  | 37 |  |  |
|  |  | LDWIATNR |  | 45 |  |  |
|  |  | PLDGNAAEAR |  | 57 |  |  |
|  |  | TDSGLEQNR |  | 58 |  |  |
|  |  | LDWIATNRV |  | 47 |  |  |
|  |  | SYFLDWIATNR |  | 79 |  |  |
|  |  | IVGGTVVEPNSLPF |  | 35 |  |  |
|  |  | IHEEYRPLTFEND |  | 62 |  |  |
|  |  | IHEEYRPLTFEND |  | [39] |  |  |
|  |  | IVGGTVVEPNSLPFQI |  | 34 |  |  |
|  |  | SGWGTTSSGGIISDELR |  | 70 |  |  |
|  |  | VVAGEHSLRTDSGLEQNR |  | 70 |  |  |
|  |  | IVGGTVVEPNSLPFQISLQR |  | 71 |  |  |
|  |  | IVGGTVVEPNSLPFQISLQR |  | [59] |  |  |
|  |  | IVGGTVVEPNSLPFQISLQR |  | [27] |  |  |
|  | 8 | AGEHSLR | 23% | 64 | [152](../../../../D:%5CDokumente%20und%20Einstellungen%5C2%5CEigene%20Dateien%5CDoktorarbeit%20Anke%5CErgebnisse%5CProteomics%5Cgeklappt%5CNIVA%20B%5C7958_7969%5C7961%20VE%5Czmmk-mascot%5Cmascot%5Ccgi%5Cprotein_view9c7f.html) | trypsin |
|  |  | GVDASILR |  | 50 |  |  |
|  |  | VVAGEHSLR |  | 75 |  |  |
|  |  | VVAGEHSLR |  | [70] |  |  |
|  |  | TDSGLEQNR |  | 58 |  |  |
|  |  | SVDVPVVDDDTCNR |  | 26 |  |  |
|  |  | SGWGTTSSGGVISDTLR |  | 40 |  |  |
|  |  | VVAGEHSLRTDSGLEQNR |  | 70 |  |  |
|  | 5 | AWLYR | 21% | 23 | [149](../../../../D:%5CDokumente%20und%20Einstellungen%5C2%5CEigene%20Dateien%5CDoktorarbeit%20Anke%5CErgebnisse%5CProteomics%5Cgeklappt%5CNIVA%20B%5C7958_7969%5C7961%20VE%5Czmmk-mascot%5Cmascot%5Ccgi%5Cprotein_viewdd6a.html) | serinprotease |
|  |  | YAWLYR |  | 25 |  |  |
|  |  | IDYDYSEER |  | 77 |  |  |
|  |  | SETCDPAEPSVFTR |  | 84 |  |  |
|  |  | TDVETTPMGTFMGWGATVAGGGFSPR |  | 43 |  |  |
|  | 4 | LDAADEPTR | 14% | 42 | [802](../../../../D:%5CDokumente%20und%20Einstellungen%5C2%5CEigene%20Dateien%5CDoktorarbeit%20Anke%5CErgebnisse%5CProteomics%5Cgeklappt%5CNIVA%20B%5C7958_7969%5C7961%20VE%5Czmmk-mascot%5Cmascot%5Ccgi%5Cprotein_view3fd9.html) | chymotrypsin |
|  |  | IINDVALIR |  | 69 |  |  |
|  |  | PSDDAAGISPVLR |  | 66 |  |  |
|  |  | LDAADEPTRVEVR |  | 26 |  |  |
|  | 6 | VSGLEQNR | 17% | 54 | [79](../../../../D:%5CDokumente%20und%20Einstellungen%5C2%5CEigene%20Dateien%5CDoktorarbeit%20Anke%5CErgebnisse%5CProteomics%5Cgeklappt%5CNIVA%20B%5C7958_7969%5C7961%20VE%5Czmmk-mascot%5Cmascot%5Ccgi%5Cprotein_view1218.html) | trypsin |
|  |  | LDLSVPSAK |  | 34 |  |  |
|  |  | IYLTTPLDLSVPSAK |  | 42 |  |  |
|  |  | IVGGTEVVPNSLPFQISLQR |  | [44] |  |  |
|  |  | IVGGTEVVPNSLPFQISLQR |  | 44 |  |  |
|  |  | IVGGTEVVPNSLPFQISLQR |  | [25] |  |  |
|  | 3 | ITETERLEIR | 16% | 29 | [448](../../../../D:%5CDokumente%20und%20Einstellungen%5C2%5CEigene%20Dateien%5CDoktorarbeit%20Anke%5CErgebnisse%5CProteomics%5Cgeklappt%5CNIVA%20B%5C7958_7969%5C7961%20VE%5Czmmk-mascot%5Cmascot%5Ccgi%5Cprotein_viewb2de.html) | chymotrypsin |
|  |  | TADGPGGISPTLQK |  | 90 |  |  |
|  |  | LSSYLSWISSITGL |  | 33 |  |  |
|  | 1 | TANEPSQVTVSTT | 5% | 46 | [383](../../../../D:%5CDokumente%20und%20Einstellungen%5C2%5CEigene%20Dateien%5CDoktorarbeit%20Anke%5CErgebnisse%5CProteomics%5Cgeklappt%5CNIVA%20B%5C7958_7969%5C7961%20VE%5Czmmk-mascot%5Cmascot%5Ccgi%5Cprotein_view1d4b.html) | chymotrypsin |
| 24 kDa | 47 | AGEHSLR | 58% | [44] | [208](../../../../D:%5CDokumente%20und%20Einstellungen%5C2%5CEigene%20Dateien%5CDoktorarbeit%20Anke%5CErgebnisse%5CProteomics%5Cgeklappt%5CNIVA%20B%5C7958_7969%5C7962%20VE%5Czmmk-mascot%5Cmascot%5Ccgi%5Cprotein_view317e.html) | trypsin |
|  |  | AGEHSLR |  | 51 |  |  |
|  |  | AGEHSLR |  | [20] |  |  |
|  |  | SGLEQNR |  | 27 |  |  |
|  |  | VVAGEHSL |  | 31 |  |  |
|  |  | GVDATILR |  | 59 |  |  |
|  |  | VAGEHSLR |  | [58] |  |  |
|  |  | VAGEHSLR |  | 67 |  |  |
|  |  | VVAGEHSLR |  | 82 |  |  |
|  |  | IVGGTVVEPN |  | 69 |  |  |
|  |  | LDWIATNR |  | 50 |  |  |
|  |  | TDSGLEQNR |  | 55 |  |  |
|  |  | TDSGLEQNR |  | [53] |  |  |
|  |  | IHEEYRPL |  | 27 |  |  |
|  |  | TDSGLEQNRG |  | 39 |  |  |
|  |  | LDWIATNRV |  | 37 |  |  |
|  |  | FLDWIATNR |  | 55 |  |  |
|  |  | IHEEYRPLT |  | 38 |  |  |
|  |  | YFLDWIATNR |  | 61 |  |  |
|  |  | SLRTDSGLEQNR |  | 31 |  |  |
|  |  | SYFLDWIATNR |  | 87 |  |  |
|  |  | SYFLDWIATNR |  | [28] |  |  |
|  |  | YFLDWIATNRV |  | 36 |  |  |
|  |  | SYFLDWIATNR |  | [57] |  |  |
|  |  | SYFLDWIATNR |  | [62] |  |  |
|  |  | IVGGTVVEPNSLPF |  | [26] |  |  |
|  |  | IVGGTVVEPNSLPF |  | [41] |  |  |
|  |  | IVGGTVVEPNSLPF |  | 44 |  |  |
|  |  | IHEEYRPLTFE |  | 30 |  |  |
|  |  | SYFLDWIATNRV |  | 47 |  |  |
|  |  | IHEEYRPLTFEN |  | 52 |  |  |
|  |  | IVGGTVVEPNSLPFQ |  | 55 |  |  |
|  |  | IVGGTVVEPNSLPFQ |  | [33] |  |  |
|  |  | TDSGLEQNRGVVSFK |  | 31 |  |  |
|  |  | IHEEYRPLTFEND |  | 35 |  |  |
|  |  | IVGGTVVEPNSLPFQI |  | 51 |  |  |
|  |  | SGWGTTSSGGIISDELR |  | 102 |  |  |
|  |  | SGWGTTSSGGIISDELR |  | [25] |  |  |
|  |  | IVGGTVVEPNSLPFQIS |  | 62 |  |  |
|  |  | SGGPLFVLPLDGNAAEAR |  | 55 |  |  |
|  |  | IHEEYRPLTFENDIS |  | 69 |  |  |
|  |  | SGWGTTSSGGIISDELRR |  | 33 |  |  |
|  |  | IVGGTVVEPNSLPFQISLQR |  | 70 |  |  |
|  |  | IVGGTVVEPNSLPFQISLQR |  | [44] |  |  |
|  |  | IHEEYRPLTFENDISLLF |  | 44 |  |  |
|  |  | IHEEYRPLTFENDISLLF |  | [22] |  |  |
|  |  | SALGSYSQSCGGSILDANVIIDAAHCVR |  | 81 |  |  |
|  | 14 | IINGAEATPH | 41% | 68 | [448](../../../../D:%5CDokumente%20und%20Einstellungen%5C2%5CEigene%20Dateien%5CDoktorarbeit%20Anke%5CErgebnisse%5CProteomics%5Cgeklappt%5CNIVA%20B%5C7958_7969%5C7962%20VE%5Czmmk-mascot%5Cmascot%5Ccgi%5Cprotein_view3eaa.html) | chymotrypsin |
|  |  | LSSYLSWISS |  | 35 |  |  |
|  |  | DVYLGAHNVR |  | 47 |  |  |
|  |  | ITETERLEIR |  | 40 |  |  |
|  |  | TADGPGGISPTLQK |  | 83 |  |  |
|  |  | LPAPVDISGNNVR |  | 54 |  |  |
|  |  | LSSYLSWISSITGL |  | 44 |  |  |
|  |  | LPAPVDISGNNVRPI |  | [47] |  |  |
|  |  | LPAPVDISGNNVRPI |  | 77 |  |  |
|  |  | IINGAEATPHEFPW |  | 61 |  |  |
|  |  | IINGAEATPHEFPWVT |  | 43 |  |  |
|  |  | YIHPDWNPNTLTGDVA |  | 40 |  |  |
|  |  | IINGAEATPHEFPWVTA |  | 48 |  |  |
|  |  | YIHPDWNPNTLTGDVALIK |  | 74 |  |  |
|  | 12 | AWLYR | 29% | 23 | [149](../../../../D:%5CDokumente%20und%20Einstellungen%5C2%5CEigene%20Dateien%5CDoktorarbeit%20Anke%5CErgebnisse%5CProteomics%5Cgeklappt%5CNIVA%20B%5C7958_7969%5C7962%20VE%5Czmmk-mascot%5Cmascot%5Ccgi%5Cprotein_viewcff0.html) | serinprotease |
|  |  | AGGGFSPR |  | 42 |  |  |
|  |  | KLDGVLR |  | 29 |  |  |
|  |  | VAGGGFSPR |  | 61 |  |  |
|  |  | YAWLYR |  | 32 |  |  |
|  |  | DYDYSEER |  | 40 |  |  |
|  |  | IDYDYSEER |  | 66 |  |  |
|  |  | SETCDPAEPSVFTR |  | 71 |  |  |
|  |  | GQVNLIDYDYSEER |  | 98 |  |  |
|  |  | AGQVNLIDYDYSEER |  | 113 |  |  |
|  |  | VLGELVVVAGQVNLIDYDYSEER |  | 26 |  |  |
|  |  | TDVETTPMGTFMGWGATVAGGGFSPR |  | 63 |  |  |
|  | 15 | AGEHSLR | 19% | [44] | [152](../../../../D:%5CDokumente%20und%20Einstellungen%5C2%5CEigene%20Dateien%5CDoktorarbeit%20Anke%5CErgebnisse%5CProteomics%5Cgeklappt%5CNIVA%20B%5C7958_7969%5C7962%20VE%5Czmmk-mascot%5Cmascot%5Ccgi%5Cprotein_view2277.html) | trypsin |
|  |  | AGEHSLR |  | 51 |  |  |
|  |  | AGEHSLR |  | [20] |  |  |
|  |  | SGLEQNR |  | 27 |  |  |
|  |  | VVAGEHSL |  | 31 |  |  |
|  |  | VAGEHSLR |  | [58] |  |  |
|  |  | VAGEHSLR |  | 67 |  |  |
|  |  | VVAGEHSLR |  | 82 |  |  |
|  |  | TDSGLEQNR |  | 55 |  |  |
|  |  | TDSGLEQNR |  | [53] |  |  |
|  |  | TDSGLEQNRG |  | 39 |  |  |
|  |  | SFFLDWIAANR |  | 28 |  |  |
|  |  | SLRTDSGLEQNR |  | 31 |  |  |
|  |  | SFFLDWIAANRG |  | 66 |  |  |
|  |  | SGWGTTSSGGVISDTLR |  | 58 |  |  |
|  | 12 | SGLEQNR | 20% | 27 | [79](../../../../D:%5CDokumente%20und%20Einstellungen%5C2%5CEigene%20Dateien%5CDoktorarbeit%20Anke%5CErgebnisse%5CProteomics%5Cgeklappt%5CNIVA%20B%5C7958_7969%5C7962%20VE%5Czmmk-mascot%5Cmascot%5Ccgi%5Cprotein_view8d39.html) | trypsin |
|  |  | VSGLEQNR |  | 51 |  |  |
|  |  | IVGGTEVVPN |  | 47 |  |  |
|  |  | SYFLDWIAANR |  | 80 |  |  |
|  |  | IVGGTEVVPNSLPF |  | 21 |  |  |
|  |  | IVGGTEVVPNSLPFQ |  | 35 |  |  |
|  |  | IVGGTEVVPNSLPFQI |  | 24 |  |  |
|  |  | IVGGTEVVPNSLPFQIS |  | 34 |  |  |
|  |  | VVAGEHDLSQVSGLEQNR |  | [51] |  |  |
|  |  | VVAGEHDLSQVSGLEQNR |  | 110 |  |  |
|  |  | IVGGTEVVPNSLPFQISLQR |  | 41 |  |  |
|  |  | IVGGTEVVPNSLPFQISLQR |  | [33] |  |  |
|  | 3 | LDAADEPTR | 9% | 45 | [802](../../../../D:%5CDokumente%20und%20Einstellungen%5C2%5CEigene%20Dateien%5CDoktorarbeit%20Anke%5CErgebnisse%5CProteomics%5Cgeklappt%5CNIVA%20B%5C7958_7969%5C7962%20VE%5Czmmk-mascot%5Cmascot%5Ccgi%5Cprotein_view3574.html) | chymotrypsin |
|  |  | IINDVALIR |  | 57 |  |  |
|  |  | LDAADEPTRVEVR |  | 31 |  |  |
|  | 3 | IVGGVEAVPH | 13% | 30 | [383](../../../../D:%5CDokumente%20und%20Einstellungen%5C2%5CEigene%20Dateien%5CDoktorarbeit%20Anke%5CErgebnisse%5CProteomics%5Cgeklappt%5CNIVA%20B%5C7958_7969%5C7962%20VE%5Czmmk-mascot%5Cmascot%5Ccgi%5Cprotein_viewe487.html) | chymotrypsin |
|  |  | FSITLGAHDR |  | 27 |  |  |
|  |  | TADGILEGVSPV |  | 31 |  |  |
| 22 kDa | 33 | TGDVALIK | 49% | 54 | [448](../../../../D:%5CDokumente%20und%20Einstellungen%5C2%5CEigene%20Dateien%5CDoktorarbeit%20Anke%5CErgebnisse%5CProteomics%5Cgeklappt%5CNIVA%20B%5C7958_7969%5C7964%20VE%5Czmmk-mascot%5Cmascot%5Ccgi%5Cprotein_viewaeeb.html) | chymotrypsin |
|  |  | LSSYLSW |  | 25 |  |  |
|  |  | YLGAHNVR |  | 39 |  |  |
|  |  | LSSYLSWI |  | 34 |  |  |
|  |  | IINGAEATPH |  | 66 |  |  |
|  |  | TLTGDVALIK |  | 42 |  |  |
|  |  | LSSYLSWIS |  | 30 |  |  |
|  |  | ITETERLEI |  | 52 |  |  |
|  |  | LSSYLSWISS |  | 47 |  |  |
|  |  | DVYLGAHNVR |  | 61 |  |  |
|  |  | TETERLEIR |  | 33 |  |  |
|  |  | TADGPGGISPTLQ |  | 34 |  |  |
|  |  | LSSYLSWISSI |  | 53 |  |  |
|  |  | ITETERLEIR |  | [32] |  |  |
|  |  | ITETERLEIR |  | 35 |  |  |
|  |  | TADGPGGISPTLQK |  | 62 |  |  |
|  |  | LPAPVDISGNNVR |  | 75 |  |  |
|  |  | YIHPDWNPNTL |  | 33 |  |  |
|  |  | SSAGCESGNPDGYAR |  | 98 |  |  |
|  |  | SSAGCESGNPDGYAR |  | [27] |  |  |
|  |  | LPAPVDISGNNVRPI |  | [60] |  |  |
|  |  | LPAPVDISGNNVRPI |  | 74 |  |  |
|  |  | LYFDVYLGAHNVR |  | 21 |  |  |
|  |  | YIHPDWNPNTLTGDV |  | 75 |  |  |
|  |  | IINGAEATPHEFPWVT |  | 55 |  |  |
|  |  | YIHPDWNPNTLTGDVA |  | 51 |  |  |
|  |  | YIHPDWNPNTLTGDVA |  | [34] |  |  |
|  |  | IINGAEATPHEFPWVTA |  | 53 |  |  |
|  |  | YIHPDWNPNTLTGDVAL |  | 45 |  |  |
|  |  | YIHPDWNPNTLTGDVAL |  | [44] |  |  |
|  |  | YIHPDWNPNTLTGDVALIK |  | 116 |  |  |
|  |  | YIHPDWNPNTLTGDVALIK |  | [23] |  |  |
|  |  | ANEKYIHPDWNPNTLTGDVALI |  | 22 |  |  |
|  | 8 | AGEHSLR | 13% | 51 | 208 | trypsin |
|  |  | VVAGEHSL |  | 29 |  |  |
|  |  | VAGEHSLR |  | [22] |  |  |
|  |  | VAGEHSLR |  | 58 |  |  |
|  |  | VVAGEHSLR |  | 79 |  |  |
|  |  | TDSGLEQNR |  | 80 |  |  |
|  |  | IVGGTVVEPNSLPFQI |  | 39 |  |  |
|  |  | IVGGTVVEPNSLPFQI |  | [20] |  |  |
|  | 7 | WIAANR | 9% | 24 | [152](../../../../D:%5CDokumente%20und%20Einstellungen%5C2%5CEigene%20Dateien%5CDoktorarbeit%20Anke%5CErgebnisse%5CProteomics%5Cgeklappt%5CNIVA%20B%5C7958_7969%5C7964%20VE%5Czmmk-mascot%5Cmascot%5Ccgi%5Cprotein_view1f35.html) | trypsin |
|  |  | AGEHSLR |  | 51 |  |  |
|  |  | VVAGEHSL |  | 29 |  |  |
|  |  | VAGEHSLR |  | [22] |  |  |
|  |  | VAGEHSLR |  | 58 |  |  |
|  |  | VVAGEHSLR |  | 79 |  |  |
|  |  | TDSGLEQNR |  | 80 |  |  |
|  | 2 | IVGGVEAVPHEFPWQVA | 16% | 30 | [383](../../../../D:%5CDokumente%20und%20Einstellungen%5C2%5CEigene%20Dateien%5CDoktorarbeit%20Anke%5CErgebnisse%5CProteomics%5Cgeklappt%5CNIVA%20B%5C7958_7969%5C7964%20VE%5Czmmk-mascot%5Cmascot%5Ccgi%5Cprotein_view08da.html) | chymotrypsin |
|  |  | TYTVHPGWNPSTLADDIALIR |  | 45 |  |  |
|  | 2 | WIAANR | 5% | 24 | [79](../../../../D:%5CDokumente%20und%20Einstellungen%5C2%5CEigene%20Dateien%5CDoktorarbeit%20Anke%5CErgebnisse%5CProteomics%5Cgeklappt%5CNIVA%20B%5C7958_7969%5C7964%20VE%5Czmmk-mascot%5Cmascot%5Ccgi%5Cprotein_view8805.html) | trypsin |
|  |  | VSGLEQNR |  | 43 |  |  |
|  | 2 | LDAADEPTR | 5% | 21 | [802](../../../../D:%5CDokumente%20und%20Einstellungen%5C2%5CEigene%20Dateien%5CDoktorarbeit%20Anke%5CErgebnisse%5CProteomics%5Cgeklappt%5CNIVA%20B%5C7958_7969%5C7964%20VE%5Czmmk-mascot%5Cmascot%5Ccgi%5Cprotein_viewa14c.html) | chymotrypsin |
|  |  | LDAADEPTRVEVR |  | 22 |  |  |
|  | 1 | DYDYSEER | 3% | 34 | [149](../../../../D:%5CDokumente%20und%20Einstellungen%5C2%5CEigene%20Dateien%5CDoktorarbeit%20Anke%5CErgebnisse%5CProteomics%5Cgeklappt%5CNIVA%20B%5C7958_7969%5C7964%20VE%5Czmmk-mascot%5Cmascot%5Ccgi%5Cprotein_view1edd.html) | trypsin |
| 21 kDa | 34 | ITETERL | 45% | 29 | [448](../../../../D:%5CDokumente%20und%20Einstellungen%5C2%5CEigene%20Dateien%5CDoktorarbeit%20Anke%5CErgebnisse%5CProteomics%5Cgeklappt%5CNIVA%20B%5C7958_7969%5C7965%20VE%5Czmmk-mascot%5Cmascot%5Ccgi%5Cprotein_viewc5f2.html) | chymotrypsin |
|  |  | RITETER |  | 33 |  |  |
|  |  | IINGAEATPH |  | 75 |  |  |
|  |  | LSSYLSWIS |  | 32 |  |  |
|  |  | LSSYLSWISS |  | 51 |  |  |
|  |  | TETERLEIR |  | 32 |  |  |
|  |  | TADGPGGISPTLQ |  | 65 |  |  |
|  |  | LSSYLSWISSI |  | 73 |  |  |
|  |  | ITETERLEIR |  | [24] |  |  |
|  |  | ITETERLEIR |  | 34 |  |  |
|  |  | ITETERLEIR |  | [27] |  |  |
|  |  | ITETERLEIR |  | [24] |  |  |
|  |  | TADGPGGISPTLQK |  | 71 |  |  |
|  |  | TADGPGGISPTLQK |  | [50] |  |  |
|  |  | LPAPVDISGNNVR |  | 85 |  |  |
|  |  | LPAPVDISGNNVR |  | [43] |  |  |
|  |  | AGCESGNPDGYAR |  | 47 |  |  |
|  |  | YIHPDWNPNTL |  | 30 |  |  |
|  |  | SSAGCESGNPDGYAR |  | 94 |  |  |
|  |  | LPAPVDISGNNVRPI |  | 83 |  |  |
|  |  | LPAPVDISGNNVRPI |  | [58] |  |  |
|  |  | VSSAGCESGNPDGYAR |  | 91 |  |  |
|  |  | IINGAEATPHEFPWVT |  | 55 |  |  |
|  |  | YIHPDWNPNTLTGDVA |  | 59 |  |  |
|  |  | YIHPDWNPNTLTGDVA |  | [37] |  |  |
|  |  | IINGAEATPHEFPWVTA |  | 66 |  |  |
|  |  | SFVSSAGCESGNPDGYAR |  | 110 |  |  |
|  |  | SFVSSAGCESGNPDGYAR |  | [71] |  |  |
|  |  | YIHPDWNPNTLTGDVAL |  | 63 |  |  |
|  |  | YIHPDWNPNTLTGDVAL |  | [40] |  |  |
|  |  | YIHPDWNPNTLTGDVALI |  | 58 |  |  |
|  |  | YIHPDWNPNTLTGDVALI |  | [28] |  |  |
|  |  | YIHPDWNPNTLTGDVALIK |  | 31 |  |  |
|  |  | IINGAEATPHEFPWVTALFI |  | 35 |  |  |
|  | 15 | IVGGVEAVPH | 40% | 58 | [383](../../../../D:%5CDokumente%20und%20Einstellungen%5C2%5CEigene%20Dateien%5CDoktorarbeit%20Anke%5CErgebnisse%5CProteomics%5Cgeklappt%5CNIVA%20B%5C7958_7969%5C7965%20VE%5Czmmk-mascot%5Cmascot%5Ccgi%5Cprotein_view8e7d.html) | chymotrypsin |
|  |  | IVGGVEAVPH |  | [29] |  |  |
|  |  | TANEPSQVTV |  | 55 |  |  |
|  |  | TLADDIALIR |  | 77 |  |  |
|  |  | TANEPSQVTVS |  | 50 |  |  |
|  |  | TADGILEGVSPV |  | 41 |  |  |
|  |  | AGCADGFPAGFTR |  | 87 |  |  |
|  |  | TANEPSQVTVSTT |  | 57 |  |  |
|  |  | VSSYSQWIADTTGL |  | 103 |  |  |
|  |  | TADGILEGVSPVLMK |  | 35 |  |  |
|  |  | TANEPSQVTVSTTTY |  | 65 |  |  |
|  |  | VSSYSQWIADTTGLI |  | 89 |  |  |
|  |  | IVGGVEAVPHEFPWQV |  | 44 |  |  |
|  |  | IVGGVEAVPHEFPWQVA |  | 46 |  |  |
|  |  | TYTVHPGWNPSTLADDIALIR |  | 72 |  |  |
|  | 7 | LDAADEPTR | 25% | 60 | [802](../../../../D:%5CDokumente%20und%20Einstellungen%5C2%5CEigene%20Dateien%5CDoktorarbeit%20Anke%5CErgebnisse%5CProteomics%5Cgeklappt%5CNIVA%20B%5C7958_7969%5C7965%20VE%5Czmmk-mascot%5Cmascot%5Ccgi%5Cprotein_view8413.html) | chymotrypsin |
|  |  | IINDVALIR |  | 77 |  |  |
|  |  | VSYFADWISSV |  | 49 |  |  |
|  |  | PSDDAAGISPVLR |  | 75 |  |  |
|  |  | LDAADEPTRVEVR |  | [26] |  |  |
|  |  | LDAADEPTRVEVR |  | 27 |  |  |
|  |  | STEYTVHPDWGPVR |  | 33 |  |  |
|  | 4 | WIAANR | 9% | 32 | [152](../../../../D:%5CDokumente%20und%20Einstellungen%5C2%5CEigene%20Dateien%5CDoktorarbeit%20Anke%5CErgebnisse%5CProteomics%5Cgeklappt%5CNIVA%20B%5C7958_7969%5C7965%20VE%5Czmmk-mascot%5Cmascot%5Ccgi%5Cprotein_view0cfa.html) | trypsin |
|  |  | VAGEHSLR |  | 39 |  |  |
|  |  | VVAGEHSLR |  | 52 |  |  |
|  |  | TDSGLEQNR |  | 48 |  |  |
|  | 3 | FDQYEATTQK | 16% | 83 | [638](../../../../D:%5CDokumente%20und%20Einstellungen%5C2%5CEigene%20Dateien%5CDoktorarbeit%20Anke%5CErgebnisse%5CProteomics%5Cgeklappt%5CNIVA%20B%5C7958_7969%5C7965%20VE%5Czmmk-mascot%5Cmascot%5Ccgi%5Cprotein_view2d24.html) | chymotrypsin |
|  |  | IVGWGATFEGGAPATR |  | [47] |  |  |
|  |  | IVGWGATFEGGAPATR |  | 75 |  |  |
| 20 kDa | 26 | IALIR | 61% | 37 | [383](../../../../D:%5CDokumente%20und%20Einstellungen%5C2%5CEigene%20Dateien%5CDoktorarbeit%20Anke%5CErgebnisse%5CProteomics%5Cgeklappt%5CNIVA%20B%5C7958_7969%5C7966%20VE%5Czmmk-mascot%5Cmascot%5Ccgi%5Cprotein_viewf5fd.html) | chymotrypsin |
|  |  | TLGAHDR |  | 52 |  |  |
|  |  | ADDIALIR |  | 71 |  |  |
|  |  | SITLGAHDR |  | 62 |  |  |
|  |  | IVGGVEAVPH |  | 70 |  |  |
|  |  | TANEPSQVTV |  | 54 |  |  |
|  |  | TLADDIALIR |  | 77 |  |  |
|  |  | FSITLGAHDR |  | [31] |  |  |
|  |  | FSITLGAHDR |  | 61 |  |  |
|  |  | TANEPSQVTVS |  | 55 |  |  |
|  |  | TADGILEGVSPV |  | 41 |  |  |
|  |  | AGCADGFPAGFTR |  | 77 |  |  |
|  |  | TANEPSQVTVSTT |  | 45 |  |  |
|  |  | TANEPSQVTVSTTT |  | 53 |  |  |
|  |  | VSSYSQWIADTTGL |  | 103 |  |  |
|  |  | TADGILEGVSPVLMK |  | 62 |  |  |
|  |  | TADGILEGVSPVLMK |  | [58] |  |  |
|  |  | TANEPSQVTVSTTTY |  | 70 |  |  |
|  |  | VSSYSQWIADTTGLI |  | 88 |  |  |
|  |  | TANEPSQVTVSTTTYT |  | 54 |  |  |
|  |  | IVGGVEAVPHEFPWQVA |  | 40 |  |  |
|  |  | IVGGVEAVPHEFPWQVA |  | [28] |  |  |
|  |  | GSCNGDSGGPLSFDNAGVY |  | 63 |  |  |
|  |  | TVHPGWNPSTLADDIALIR |  | 80 |  |  |
|  |  | CLAPSTESNHVGDTLLVSGWGK |  | 55 |  |  |
|  |  | TYTVHPGWNPSTLADDIALIR |  | 38 |  |  |
|  | 25 | ITETERL | 43% | 36 | [448](../../../../D:%5CDokumente%20und%20Einstellungen%5C2%5CEigene%20Dateien%5CDoktorarbeit%20Anke%5CErgebnisse%5CProteomics%5Cgeklappt%5CNIVA%20B%5C7958_7969%5C7966%20VE%5Czmmk-mascot%5Cmascot%5Ccgi%5Cprotein_view05d7.html) | chymotrypsin |
|  |  | IINGAEATPH |  | 84 |  |  |
|  |  | TLTGDVALIK |  | 22 |  |  |
|  |  | DVYLGAHNVR |  | 58 |  |  |
|  |  | TETERLEIR |  | 23 |  |  |
|  |  | TADGPGGISPTLQ |  | 43 |  |  |
|  |  | ITETERLEIR |  | 54 |  |  |
|  |  | GCESGNPDGYAR |  | 33 |  |  |
|  |  | TADGPGGISPTLQK |  | 73 |  |  |
|  |  | TADGPGGISPTLQK |  | [50] |  |  |
|  |  | LPAPVDISGNNVR |  | 83 |  |  |
|  |  | AGCESGNPDGYAR |  | 30 |  |  |
|  |  | YIHPDWNPNTL |  | 30 |  |  |
|  |  | SSAGCESGNPDGYAR |  | 85 |  |  |
|  |  | LPAPVDISGNNVRPI |  | 79 |  |  |
|  |  | VSSAGCESGNPDGYAR |  | 86 |  |  |
|  |  | IINGAEATPHEFPWVT |  | 37 |  |  |
|  |  | YIHPDWNPNTLTGDVA |  | 21 |  |  |
|  |  | IINGAEATPHEFPWVTA |  | 55 |  |  |
|  |  | SFVSSAGCESGNPDGYAR |  | 110 |  |  |
|  |  | SFVSSAGCESGNPDGYAR |  | [70] |  |  |
|  |  | YIHPDWNPNTLTGDVALI |  | [37] |  |  |
|  |  | YIHPDWNPNTLTGDVALI |  | 41 |  |  |
|  |  | YIHPDWNPNTLTGDVALIK |  | 86 |  |  |
|  |  | YIHPDWNPNTLTGDVALIK |  | [51] |  |  |
|  | 9 | IVGGTEAVPH | 29% | 72 | [802](../../../../D:%5CDokumente%20und%20Einstellungen%5C2%5CEigene%20Dateien%5CDoktorarbeit%20Anke%5CErgebnisse%5CProteomics%5Cgeklappt%5CNIVA%20B%5C7958_7969%5C7966%20VE%5Czmmk-mascot%5Cmascot%5Ccgi%5Cprotein_view95c2.html) | chymotrypsin |
|  |  | IINDVALIR |  | 67 |  |  |
|  |  | AADEPTRVEVR |  | 21 |  |  |
|  |  | VSYFADWISSV |  | 56 |  |  |
|  |  | PSDDAAGISPVLR |  | 91 |  |  |
|  |  | LDAADEPTRVEVR |  | 34 |  |  |
|  |  | LDAADEPTRVEVR |  | [24] |  |  |
|  |  | STEYTVHPDWGPVR |  | [32] |  |  |
|  |  | STEYTVHPDWGPVR |  | 50 |  |  |
|  | 5 | FDQYEATTQK | 35% | 84 | [638](../../../../D:%5CDokumente%20und%20Einstellungen%5C2%5CEigene%20Dateien%5CDoktorarbeit%20Anke%5CErgebnisse%5CProteomics%5Cgeklappt%5CNIVA%20B%5C7958_7969%5C7966%20VE%5Czmmk-mascot%5Cmascot%5Ccgi%5Cprotein_view7b14.html) | chymotrypsin |
|  |  | GWGATFEGGAPATR |  | 86 |  |  |
|  |  | IVGWGATFEGGAPATR |  | 85 |  |  |
|  |  | QDQHICGGFIYNDR |  | 26 |  |  |
|  |  | RYDEIDELAQPWEAK |  | 37 |  |  |
|  | 1 | IVGGTEAVPN | 4% | 45 | [460](../../../../D:%5CDokumente%20und%20Einstellungen%5C2%5CEigene%20Dateien%5CDoktorarbeit%20Anke%5CErgebnisse%5CProteomics%5Cgeklappt%5CNIVA%20B%5C7958_7969%5C7966%20VE%5Czmmk-mascot%5Cmascot%5Ccgi%5Cprotein_viewa733.html) | trypsin |
| 19 kDa | 29 | LGSHNVR | 65% | 22 | [802](../../../../D:%5CDokumente%20und%20Einstellungen%5C2%5CEigene%20Dateien%5CDoktorarbeit%20Anke%5CErgebnisse%5CProteomics%5Cgeklappt%5CNIVA%20B%5C7958_7969%5C7967%20VE%5Czmmk-mascot%5Cmascot%5Ccgi%5Cprotein_view786e.html) | chymotrypsin |
|  |  | NDVALIR |  | 38 |  |  |
|  |  | IINDVALI |  | 41 |  |  |
|  |  | LLGSHNVR |  | 52 |  |  |
|  |  | LDAADEPTR |  | 60 |  |  |
|  |  | VSYFADWI |  | 41 |  |  |
|  |  | IINDVALIR |  | [30] |  |  |
|  |  | IINDVALIR |  | 71 |  |  |
|  |  | IINDVALIR |  | [24] |  |  |
|  |  | STEYTVHPD |  | 27 |  |  |
|  |  | VSYFADWIS |  | 36 |  |  |
|  |  | NILLGSHNVR |  | 55 |  |  |
|  |  | NILLGSHNVR |  | [27] |  |  |
|  |  | GSCNGDSGGPLSF |  | 67 |  |  |
|  |  | VSYFADWISSV |  | 68 |  |  |
|  |  | PSDDAAGISPVLR |  | 94 |  |  |
|  |  | PSDDAAGISPVLR |  | [43] |  |  |
|  |  | LDAADEPTRVEVR |  | 47 |  |  |
|  |  | SSAGCEVGLPAGFAR |  | 77 |  |  |
|  |  | GKPSDDAAGISPVLR |  | [25] |  |  |
|  |  | GKPSDDAAGISPVLR |  | 31 |  |  |
|  |  | GSSAGCEVGLPAGFAR |  | 83 |  |  |
|  |  | LPNPIEFTPEIQPI |  | 58 |  |  |
|  |  | STEYTVHPDWGPVR |  | 56 |  |  |
|  |  | STEYTVHPDWGPVR |  | [32] |  |  |
|  |  | VSYFADWISSVTGLV |  | 80 |  |  |
|  |  | IVGGTEAVPHSAPWQVA |  | 38 |  |  |
|  |  | SFGSSAGCEVGLPAGFAR |  | 61 |  |  |
|  |  | EVDVPCISNAECADTY |  | 50 |  |  |
|  | 19 | TLGAHDR | 58% | 40 | [383](../../../../D:%5CDokumente%20und%20Einstellungen%5C2%5CEigene%20Dateien%5CDoktorarbeit%20Anke%5CErgebnisse%5CProteomics%5Cgeklappt%5CNIVA%20B%5C7958_7969%5C7967%20VE%5Czmmk-mascot%5Cmascot%5Ccgi%5Cprotein_view9d84.html) | chymotrypsin |
|  |  | SITLGAHDR |  | 63 |  |  |
|  |  | IVGGVEAVPH |  | 84 |  |  |
|  |  | FSITLGAHDR |  | 71 |  |  |
|  |  | TANEPSQVTVS |  | 39 |  |  |
|  |  | GSCNGDSGGPLSF |  | 67 |  |  |
|  |  | AGCADGFPAGFTR |  | 68 |  |  |
|  |  | TANEPSQVTVSTT |  | 61 |  |  |
|  |  | LPSPVAFTPEIAPI |  | 47 |  |  |
|  |  | VSSYSQWIADTTGL |  | 90 |  |  |
|  |  | TADGILEGVSPVLMK |  | [71] |  |  |
|  |  | TADGILEGVSPVLMK |  | 86 |  |  |
|  |  | TADGILEGVSPVLMK |  | [54] |  |  |
|  |  | TANEPSQVTVSTTTY |  | 73 |  |  |
|  |  | TANEPSQVTVSTTTYT |  | 66 |  |  |
|  |  | IVGGVEAVPHEFPWQVA |  | 38 |  |  |
|  |  | GSCNGDSGGPLSFDNAGVY |  | 50 |  |  |
|  |  | TVHPGWNPSTLADDIALIR |  | [53] |  |  |
|  |  | TVHPGWNPSTLADDIALIR |  | 97 |  |  |
|  | 5 | IINGAEATPH | 27% | 57 | [448](../../../../D:%5CDokumente%20und%20Einstellungen%5C2%5CEigene%20Dateien%5CDoktorarbeit%20Anke%5CErgebnisse%5CProteomics%5Cgeklappt%5CNIVA%20B%5C7958_7969%5C7967%20VE%5Czmmk-mascot%5Cmascot%5Ccgi%5Cprotein_viewea08.html) | chymotrypsin |
|  |  | TADGPGGISPTLQK |  | 88 |  |  |
|  |  | LPAPVDISGNNVRPI |  | 67 |  |  |
|  |  | IINGAEATPHEFPWVTA |  | 50 |  |  |
|  |  | YIHPDWNPNTLTGDVALIK |  | 50 |  |  |
|  | 1 | IVGGTEAVPN | 4% | 63 | [460](../../../../D:%5CDokumente%20und%20Einstellungen%5C2%5CEigene%20Dateien%5CDoktorarbeit%20Anke%5CErgebnisse%5CProteomics%5Cgeklappt%5CNIVA%20B%5C7958_7969%5C7967%20VE%5Czmmk-mascot%5Cmascot%5Ccgi%5Cprotein_view407d.html) | trypsin |
| 18 kDa | 47 | IINDVALI | 62% | 28 | [802](../../../../D:%5CDokumente%20und%20Einstellungen%5C2%5CEigene%20Dateien%5CDoktorarbeit%20Anke%5CErgebnisse%5CProteomics%5Cgeklappt%5CNIVA%20B%5C7958_7969%5C7968%20VE%5Czmmk-mascot%5Cmascot%5Ccgi%5Cprotein_view7304.html) | chymotrypsin |
|  |  | AAGISPVLR |  | 40 |  |  |
|  |  | LLGSHNVR |  | [45] |  |  |
|  |  | LLGSHNVR |  | 64 |  |  |
|  |  | LDAADEPTR |  | 53 |  |  |
|  |  | LDAADEPTR |  | [30] |  |  |
|  |  | LDAADEPTR |  | [21] |  |  |
|  |  | LDAADEPTR |  | [49] |  |  |
|  |  | DAAGISPVLR |  | 56 |  |  |
|  |  | VSYFADWI |  | 57 |  |  |
|  |  | IINDVALIR |  | [73] |  |  |
|  |  | IINDVALIR |  | [35] |  |  |
|  |  | IINDVALIR |  | [70] |  |  |
|  |  | IINDVALIR |  | [26] |  |  |
|  |  | IINDVALIR |  | 80 |  |  |
|  |  | IINDVALIR |  | [62] |  |  |
|  |  | IINDVALIR |  | [60] |  |  |
|  |  | STEYTVHPD |  | 44 |  |  |
|  |  | GNICVDTTGGK |  | 63 |  |  |
|  |  | NILLGSHNVR |  | 34 |  |  |
|  |  | NILLGSHNVR |  | [29] |  |  |
|  |  | TVHPDWGPVR |  | 35 |  |  |
|  |  | AADEPTRVEVR |  | 25 |  |  |
|  |  | AADEPTRVEVR |  | [21] |  |  |
|  |  | GSCNGDSGGPLSF |  | 83 |  |  |
|  |  | VSYFADWISSV |  | 72 |  |  |
|  |  | PSDDAAGISPVLR |  | 96 |  |  |
|  |  | PSDDAAGISPVLR |  | [62] |  |  |
|  |  | LDAADEPTRVEVR |  | 39 |  |  |
|  |  | LDAADEPTRVEVR |  | [31] |  |  |
|  |  | SSAGCEVGLPAGFAR |  | 105 |  |  |
|  |  | GKPSDDAAGISPVLR |  | 71 |  |  |
|  |  | GSSAGCEVGLPAGFAR |  | 79 |  |  |
|  |  | VSYFADWISSVTGL |  | [47] |  |  |
|  |  | VSYFADWISSVTGL |  | [50] |  |  |
|  |  | VSYFADWISSVTGL |  | 70 |  |  |
|  |  | LPNPIEFTPEIQPI |  | 66 |  |  |
|  |  | STEYTVHPDWGPVR |  | [20] |  |  |
|  |  | STEYTVHPDWGPVR |  | 43 |  |  |
|  |  | VSYFADWISSVTGLV |  | 88 |  |  |
|  |  | VSYFADWISSVTGLV |  | [28] |  |  |
|  |  | VSYFADWISSVTGLV |  | [72] |  |  |
|  |  | IVGGTEAVPHSAPWQV |  | 50 |  |  |
|  |  | VSYFADWISSVTGLV |  | [39] |  |  |
|  |  | VSYFADWISSVTGLV |  | [74] |  |  |
|  |  | STEYTVHPDWGPVR |  | [31] |  |  |
|  |  | IVGGTEAVPHSAPWQVA |  | 58 |  |  |
|  | 6 | GSCNGDSGGPLSF | 32% | 83 | [383](../../../../D:%5CDokumente%20und%20Einstellungen%5C2%5CEigene%20Dateien%5CDoktorarbeit%20Anke%5CErgebnisse%5CProteomics%5Cgeklappt%5CNIVA%20B%5C7958_7969%5C7968%20VE%5Czmmk-mascot%5Cmascot%5Ccgi%5Cprotein_viewc610.html) | chymotrypsin |
|  |  | VSSYSQWIADTTGL |  | 87 |  |  |
|  |  | TADGILEGVSPVLMK |  | 71 |  |  |
|  |  | TADGILEGVSPVLMK |  | [60] |  |  |
|  |  | TANEPSQVTVSTTTY |  | 76 |  |  |
|  |  | TVHPGWNPSTLADDIALIR |  | 29 |  |  |
|  | 2 | TADGPGGISPTLQK | 13% | 88 | [448](../../../../D:%5CDokumente%20und%20Einstellungen%5C2%5CEigene%20Dateien%5CDoktorarbeit%20Anke%5CErgebnisse%5CProteomics%5Cgeklappt%5CNIVA%20B%5C7958_7969%5C7968%20VE%5Czmmk-mascot%5Cmascot%5Ccgi%5Cprotein_view4737.html) | chymotrypsin |
|  |  | YIHPDWNPNTLTGDVALIK |  | 33 |  |  |
|  | 1 | IVGGTEAVPN | 4% | 33 | [460](../../../../D:%5CDokumente%20und%20Einstellungen%5C2%5CEigene%20Dateien%5CDoktorarbeit%20Anke%5CErgebnisse%5CProteomics%5Cgeklappt%5CNIVA%20B%5C7958_7969%5C7968%20VE%5Czmmk-mascot%5Cmascot%5Ccgi%5Cprotein_view7e93.html) | trypsin |
| 17 kDa | 20 | LLGSHNVR | 43% | 55 | [802](../../../../D:%5CDokumente%20und%20Einstellungen%5C2%5CEigene%20Dateien%5CDoktorarbeit%20Anke%5CErgebnisse%5CProteomics%5Cgeklappt%5CNIVA%20B%5C7958_7969%5C7969%20VE%5Czmmk-mascot%5Cmascot%5Ccgi%5Cprotein_view87f1.html) | chymotrypsin |
|  |  | LDAADEPTR |  | [40] |  |  |
|  |  | LDAADEPTR |  | 53 |  |  |
|  |  | LDAADEPTR |  | [25] |  |  |
|  |  | IINDVALIR |  | 71 |  |  |
|  |  | IINDVALIR |  | [65] |  |  |
|  |  | NILLGSHNVR |  | 21 |  |  |
|  |  | GSCNGDSGGPLSF |  | 41 |  |  |
|  |  | PSDDAAGISPVLR |  | [48] |  |  |
|  |  | PSDDAAGISPVLR |  | 90 |  |  |
|  |  | PSDDAAGISPVLR |  | [50] |  |  |
|  |  | LDAADEPTRVEVR |  | [32] |  |  |
|  |  | LDAADEPTRVEVR |  | 40 |  |  |
|  |  | GSSAGCEVGLPAGFAR |  | 78 |  |  |
|  |  | VSYFADWISSVTGL |  | [52] |  |  |
|  |  | VSYFADWISSVTGL |  | 77 |  |  |
|  |  | STEYTVHPDWGPVR |  | 37 |  |  |
|  |  | VSYFADWISSVTGLV |  | [70] |  |  |
|  |  | VSYFADWISSVTGLV |  | [75] |  |  |
|  |  | VSYFADWISSVTGLV |  | 105 |  |  |
|  | 3 | FSITLGAHDR | 16% | 22 | [383](../../../../D:%5CDokumente%20und%20Einstellungen%5C2%5CEigene%20Dateien%5CDoktorarbeit%20Anke%5CErgebnisse%5CProteomics%5Cgeklappt%5CNIVA%20B%5C7958_7969%5C7969%20VE%5Czmmk-mascot%5Cmascot%5Ccgi%5Cprotein_view8b50.html) | chymotrypsin |
|  |  | GSCNGDSGGPLSF |  | 41 |  |  |
|  |  | TADGILEGVSPVLMK |  | 37 |  |  |
|  | 1 | TADGPGGISPTLQK | 5% | 58 | [448](../../../../D:%5CDokumente%20und%20Einstellungen%5C2%5CEigene%20Dateien%5CDoktorarbeit%20Anke%5CErgebnisse%5CProteomics%5Cgeklappt%5CNIVA%20B%5C7958_7969%5C7969%20VE%5Czmmk-mascot%5Cmascot%5Ccgi%5Cprotein_view93f2.html) | chymotrypsin |
